# Analysis of the contributing role of drug transport across biological barriers in the development and treatment of chemotherapy-induced peripheral neuropathy

**DOI:** 10.1186/s12987-024-00519-7

**Published:** 2024-02-08

**Authors:** Yang Hu, Milda Girdenyté, Lieke Roest, Iida Liukkonen, Maria Siskou, Frida Bällgren, Margareta Hammarlund-Udenaes, Irena Loryan

**Affiliations:** 1 Translational Pharmacokinetics-Pharmacodynamics Group, tPKPD, Department of Pharmacy, Faculty of Pharmacy, Uppsala University, Box 580, 751 23 Uppsala, Sweden; 2 Current Affiliation: Discovery ADME, Drug Discovery Sciences, Boehringer Ingelheim RCV, GmbH & Co KG, 1121 Vienna, Austria; 3 Pharmacy and Pharmacology Center, Institute of Biomedical Sciences, Faculty of Medicine, Vilnius University, M.K. Čiurlionio, Str. 21/27, 03101 Vilnius, Lithuania

**Keywords:** Chemotherapy-induced peripheral neuropathy, CIPN, Blood–nerve barrier, Blood–dorsal root ganglion barrier, K_p,uu_, K_p,uu,cell_, Neuropharmacokinetics

## Abstract

**Background:**

Chemotherapy-induced peripheral neuropathy (CIPN) represents a major unmet medical need that currently has no preventive and/or curative treatment. This is, among others, driven by a poor understanding of the contributive role of drug transport across biological barriers to target-site exposure.

**Methods:**

Here, we systematically investigated the transport of 11 small-molecule drugs, both, associated and not with CIPN development, at conventional (dorsal root ganglia, sciatic nerve) and non-conventional (brain, spinal cord, skeletal muscle) CIPN sites. We developed a Combinatory Mapping Approach for CIPN, CMA-CIPN, combining in vivo and in vitro elements.

**Results:**

Using CMA-CIPN, we determined the unbound tissue-to-plasma concentration ratio (K_p,uu_) and the unbound intracellular-to-extracellular concentration ratio (K_p,uu,cell_), to quantitatively assess the extent of unbound drug transport across endothelial interfaces and parenchymal cellular barriers of investigated CIPN-sites, respectively, in a rat model. The analysis revealed that unique pharmacokinetic characteristics underly time-dependent accumulation of the CIPN-positive drugs paclitaxel and vincristine at conventional (dorsal root ganglia and sciatic nerve) and non-conventional (skeletal muscle) CIPN sites. Investigated CIPN-positive drugs displayed intracellular accumulation contrary to CIPN-negative drugs nilotinib and methotrexate, which lacked this feature in all investigated tissues.

**Conclusions:**

Hence, high unbound drug intracellular and extracellular exposure at target sites, driven by an interplay of drug transport across the endothelial and parenchymal cellular barriers, is a predisposing factor to CIPN development for CIPN-positive drugs. Critical drug-specific features of unbound drug disposition at various CIPN- sites provide invaluable insights into understanding the pharmacological/toxicological effects at the target-sites which will inform new strategies for monitoring and treatment of CIPN.

**Supplementary Information:**

The online version contains supplementary material available at 10.1186/s12987-024-00519-7.

## Background

Chemotherapy-induced peripheral neuropathy (CIPN) is a dose‐limiting adverse effect commonly experienced by cancer patients after chemotherapy [1–4]. CIPN-causing drugs (“CIPN-D”) represent various pharmacological classes, including taxanes, vinca alkaloids, platins, and proteasome inhibitors [5]. Even though CIPN is a subject of intense research, no preventive and/or curative treatment is currently available [1]. That may be because of a poor understanding of CIPN-D exposure at the target anatomical site(s), and the lack of systematic characterization of CIPN-D exposure–response relationships.

Initially considered to be an exclusively peripheral phenomenon, CIPN develops at various sites in the body. The so-called “CIPN conventional sites” are the dorsal root ganglia (DRG) and the distal nerve terminals in the peripheral nervous system (PNS) [2], while the “CIPN non-conventional sites” are the central nervous system (CNS), e.g., the brain (Br) and spinal cord (SC), and, possibly, muscles [6–8]. Different pathophysiological mechanisms underlying CIPN have been proposed at several of these sites. For instance, nucleolar abnormalities in the DRG have been observed following the administration of taxanes and vinca alkaloids [9]. Further, primary axonopathy, nerve degeneration, sensory fiber demyelination, and reduction of the blood supply via *vasa nervorum* were reported for taxane use [10, 11]. In the CNS, CIPN can be associated with hyperactivity and hyperexcitability in several brain regions, reduced GABA-ergic inhibition in the brain, and a pro-inflammatory state [7]. In addition, accumulated clinical evidence indicates chronic skeletal muscle (SM) toxicity of chemotherapy [8, 12]. Yet, there is a lack of detailed mechanistic investigations on the possible impact of CIPN-D on skeletal muscle and its association with CIPN pathology. The complexity and multi-tissue involvement of CIPN pathophysiology suggest differences in tissue-specific exposure of CIPN-D. However, to date, this has not been systematically investigated and the exact mechanisms involved in the development of CIPN remain elusive.

Although no unified guidelines exist for the prevention and treatment of CIPN in clinical practice, many progressive oncological centers perform plasma drug exposure-guided cancer treatment with CIPN-D, e.g., [3, 13, 14]. However, plasma exposure does not necessarily reflect the concentrations at the key anatomical CIPN sites [15]. Also, some preclinical studies revealed the accumulation and retention of chemotherapeutic drugs at CIPN sites that may trigger pathological sequelae leading to CIPN manifestation [16]. Consequently, an improved understanding of the relationship between plasma and CIPN site exposure is imperative. However, systematic in vivo preclinical investigations of the pharmacokinetics (PK) at conventional and non-conventional CIPN sites to address this are lacking.

**Fig. 1:**
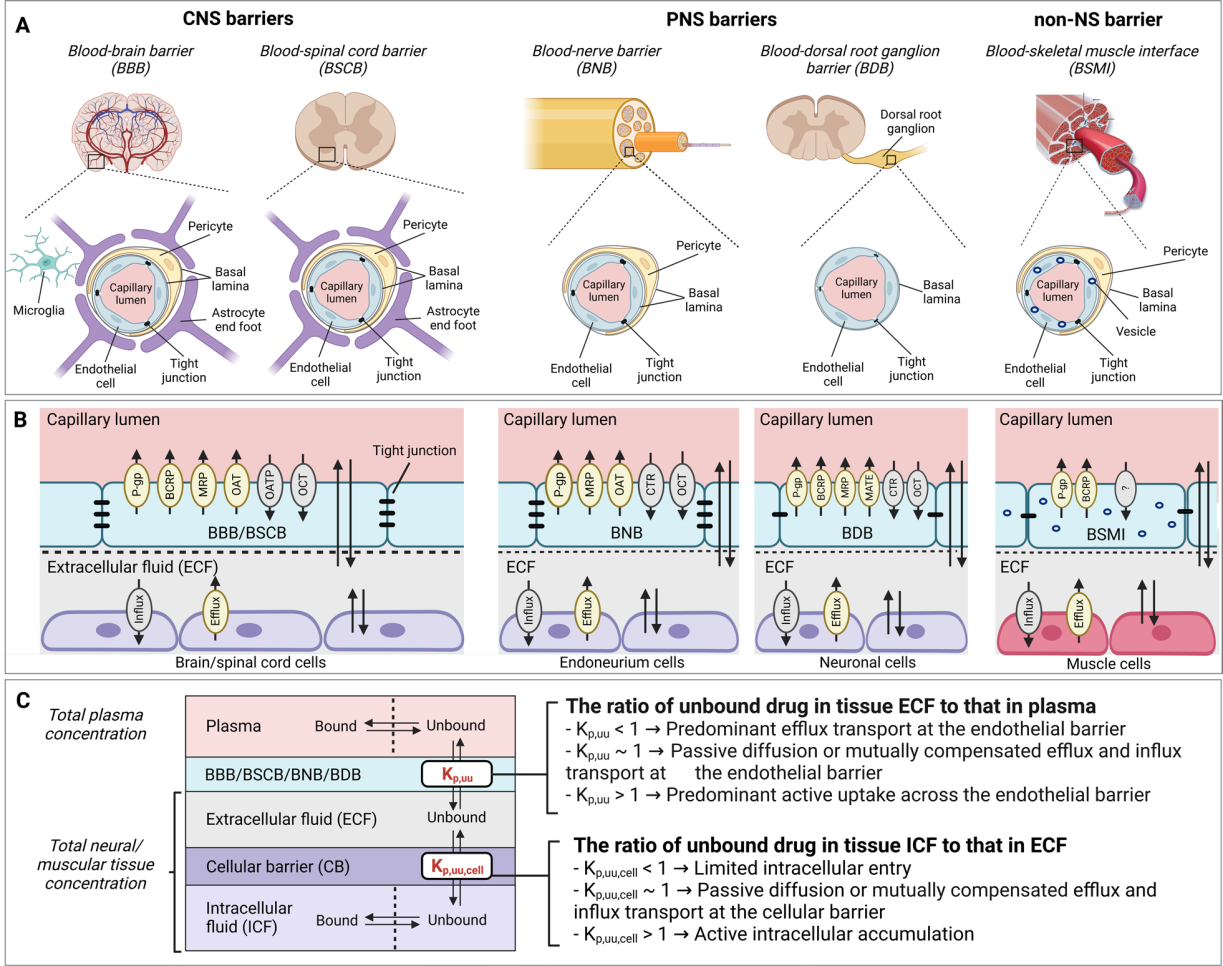
Anatomical and functional differences between the CNS, PNS, and SM (non-NS) barriers, with an overview of key PK parameters for the evaluation of unbound drug exposure. **A** Morphological structures of neurovascular and muscle microvascular units at the different biological barriers. The structure of the BNB is similar to that of the BBB and BSCB, except that it lacks astrocytes. Compared to the BNB, the neuron-rich region in the BDB lacks pericyte coverage and is leakier. The BSMI structure is similar to that of the BNB, except for the pronounced vesicular transport in endothelial cells. **B** Expression of influx and efflux membrane transporters on the endothelial and parenchymal cellular barriers. For the illustration purposes, only representative transporters are indicated on the luminal side of the endothelial cells (not necessarily their actual location). Further, only representative influx and efflux transporters are shown at the cellular membrane since the expression of specific transporters is often not reported. The passive diffusion is indicated by two parallel arrows pointing in opposite directions. **C** The extent of unbound drug transport across the biological barriers is characterized by the unbound tissue extracellular-to-plasma concentration ratio (K_p,uu_) and unbound tissue intracellular-to-extracellular concentration ratio (K_p,uu,cell_)

A few preclinical studies have reported PNS distribution of typical CIPN-Ds [17–19]. However, these studies only measured total drug concentrations in the plasma and PNS tissues, such as the DRG and sciatic nerve (SN). This is a major limitation as it is well recognized that only the unbound drug crosses biological barriers, engages with the extracellular or intracellular target(s), and exerts a pharmacological or toxicological effect [20]. Further, drug disposition at CIPN sites is complex and involves multiple interdependent processes, including passage across primary endothelial barriers, e.g., the blood–dorsal root ganglion barrier (BDB), blood–nerve barrier (BNB), blood–brain barrier (BBB), blood–spinal cord barrier (BSCB), and blood–skeletal muscle interface/barrier (BSMI), followed by the passage across the secondary parenchymal cellular barriers (Fig. 1). One solution to evaluate CIPN-D disposition at CIPN sites is to use a combinatory mapping approach (CMA) [21–23]. The original CMA, which includes in vivo neuroPK, in vitro brain slice and in vitro equilibrium dialysis, allows the assessment of key PK parameters, i.e., unbound tissue (extracellular)-to-plasma concentration ratio (K_p,uu_) for the characterization of the extent of BBB transport, and the unbound intracellular-to-extracellular concentration ratio (K_p,uu,cell_) characterizing the extent of brain parenchymal cellular barrier (CB) transport (Fig. 1C). However, this approach had not been extended and adapted in the CIPN context to date.

In the current study, we investigated the contributive role of drug transport at the biological barriers to target-site exposure, focusing on the extracellular and intracellular disposition of toxicologically and pharmacologically relevant entities of drugs. We examined both, drugs that are associated (CIPN-positive) and not (CIPN-negative) with CIPN development, and chemotherapeutic as well as non-chemotherapeutic agents. We systematically evaluated the extent of their transport across PNS, CNS, and SM endothelial barriers, and parenchymal CB at various CIPN sites in a rat model. Using CMA-CIPN, a new methodology developed here as an extension of CMA, we assessed the key PK parameters K_p,uu_ and K_p,uu,cell_ in five tissues representing conventional and non-conventional CIPN sites. The analysis revealed unique tissue distribution properties of CIPN-positive drugs that predispose them to accumulate at conventional CIPN sites and SM. The important differences in unbound drug disposition to various CIPN-target tissues underly the complexity of the relationships between target-site exposure and potential pharmacological/toxicological response. These findings will inform new strategies on how to monitor plasma exposure in patients during chemotherapy and also facilitate the development of novel translatable approaches to prevent or mitigate CIPN.

## Methods

### Study overview

In the study, the extent of drug transport across the blood-to-tissue barriers and parenchymal CB was evaluated using the PK parameters K_p,uu_ and K_p,uu,cell_, respectively (Fig. 1C), in a rat model. This was done using a new approach, CMA-CIPN. CMA-CIPN consists of in vivo PK studies and a set of in vitro assays for the evaluation of drug disposition at conventional and non-conventional CIPN-sites. All the analytes were analyzed by ultra-performance liquid chromatography–tandem mass spectrometry (UPLC-MS/MS). The experimental details are presented in Additional file 3, with an overview provided below.

### Tested drugs

The extent of transport across the PNS, CNS, and SM endothelial barriers, including the BNB, BDB, BBB, BSCB, and BSMI, as well as parenchymal CB at various CIPN sites was systematically examined for a set of 11 small-molecular-weight drugs (Table 1, Additional file 2: Table S1). The set included CIPN-positive and CIPN-negative drugs, as well as non-chemotherapeutic agents associated with the development of peripheral neuropathy (PN), i.e., PN-positive compounds, to explore whether the PK patterns of PN-inducing drugs share similarities with those of CIPN-Ds, and PN-negative drugs. The selection of PN-negative drugs was driven by two main criteria: (i) potential role in the symptomatic treatment or prevention of CIPN/PN [e.g., [24–28]]; and (ii) known differences in the extent of transport across biological membranes governed by specific transporters (Additional file 2: Table S1).

**Table 1:** Overview of pharmacology and PN-centered toxicology of the selected drugs

Category	Compound	Pharmacology		Localization of a pharmacological target	Toxicology (PN)	
		Class	Pharmacological target		Positive or negative	Target site
CIPN-positive or negative	Paclitaxel	Taxane	Tubulin	Intracellular	Positive	DRG, axon, and distal nerve termina [48]
	Vincristine	Vinca alkaloid	Tubulin	Intracellular	Positive	DRG, axon [49]
	Methotrexate	Antimetabolite	Dihydrofolate reductase Thymidylate synthase Bifunctional purine biosynthesis protein	Intracellular	Negative	N.A
	Nilotinib	Tyrosine kinase inhibitor	Tyrosine-protein kinase ABL-1^§^ Mast/stem cell growth factor receptor	Intracellular^§^	Negative	N.A
PN-positive or negative	Isoniazid	Antitubercular agent	Long-chain enoyl-acyl carrier protein reductase (InhA) Pyridoxal kinase^a^	Intracellular	Positive	Nerve terminals [50]
	Acrylamide	N.A	N.A		Positive	Nerve terminals [51]
	Varenicline	Smoking cessation aid	α4β2 neuronal nicotinic acetylcholine receptor	Extracellular	Negative	N.A
	Oxycodone	Opioid analgesic	Opioid receptors	Extracellular	Negative	N.A
	Paroxetine	Selective serotonin-reuptake inhibitor	Serotonin reuptake transporter	Extracellular	Negative	N.A
	Monomethyl fumarate	Immunomodulatory drug	Nuclear factor erythroid 2-related factor 2	Intracellular	Negative	N.A
	Diazepam	Benzodiazepine	GABA receptor	Extracellular	Negative	N.A

^a^Toxicological target

^§^localization is listed only for Tyrosine-protein kinase ABL-1

*N.A.* not applicable, *N.I.* not identified

### Animal model and permissions

Male Sprague–Dawley rats (n = 91 in total) from Taconic (Lille Skensved, Denmark) or male Wistar-Han rats (n = 17 in total) from Charles River Laboratories, Inc. (Germany) were used for all the experiments. Before experiments, the rats were housed in groups and acclimatized for seven days under temperature- and humidity-controlled conditions in a 12 h light/dark cycle with unlimited access to food and water. The rats weighed 240–340 g on the day of the experiments. All the experimental protocols and animal procedures were approved by the Uppsala Regional Animal Ethics Committee (Dnr 5.8.18–12,230/2019, Uppsala, Sweden) and were performed at the Department of Pharmacy, Biomedical Centre, Uppsala University (Husargatan 3, 751 23 Uppsala, Sweden). Animal studies have been reported in agreement with ARRIVE (Animal Research: Reporting of In Vivo Experiments) guidelines. All studies were non-randomized and non-blinded. A priori estimated minimally required per group sample size for a two-tailed t-test study was six to four rats, given the probability level of α 0.05, the anticipated effect size, i.e., Cohen’s d in the range of 2 to 2.5, and the desired statistical power level of 0.8. The selection of only male rats was based on the intention to minimize inter-subject variability in the determined PK parameters, which impacts the sample size estimation.

### In vivo PK studies to assess total drug distribution into CIPN sites

**Fig. 2:**
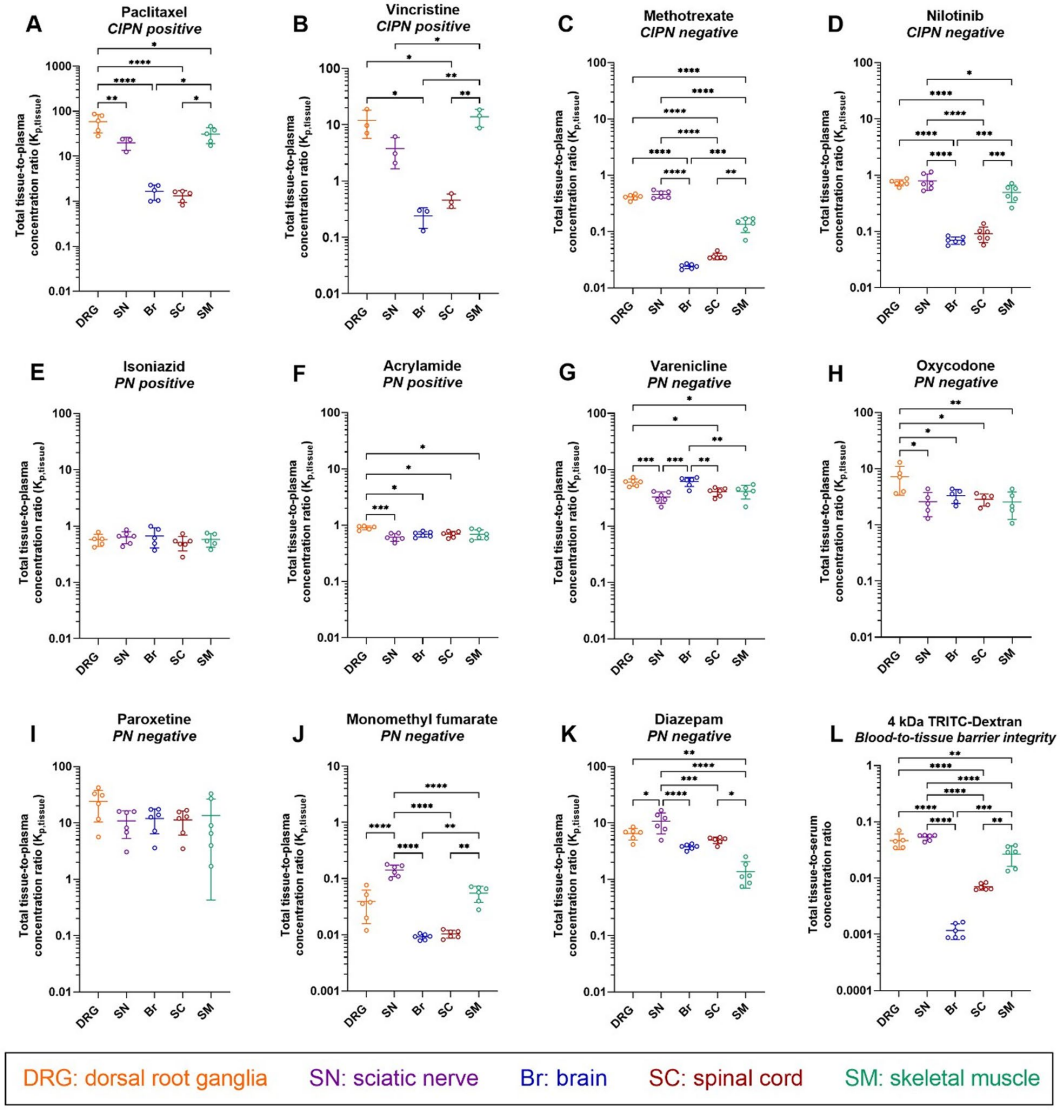
Scatter dot plots of the total tissue-to-plasma concentration ratio (K_p,tissue_) determined at steady-state for the indicated drugs and the total tissue-to-serum concentration ratio of 4 kDa TRITC-dextran in the indicated tissues. **A–K** Measurements for the different drugs, with their CIPN and PN propensity indicated. **L** Measurements for 4 kDa TRITC-dextran. Data are shown as the mean ± SD, n = 3–6 biological replicates. *p < 0.05, **p < 0.01, ***p < 0.001, or ****p < 0.0001 (ordinary one-way ANOVA with Tukey’s multiple comparison test). NB: note the differences in scale

To assess total drug partitioning to CIPN sites in vivo, total tissue-to-plasma concentration ratio (K_p,tissue_) was determined under steady-state conditions following a 4-h constant intravenous (IV) infusion (n = 3–6 animals per drug). The dosing regimen of each drug was designed based on systemic rat PK parameters and clinically relevant therapeutic unbound steady-state plasma concentration estimated based on total concentrations and fraction of unbound drug in plasma reported in humans (Additional file 2: Tables S2 and S3). Considering the observed slow tissue distribution and the reported non-parallel PK profiles between plasma and CIPN sites for paclitaxel and vincristine [18], 4-h IV infusion regimen was found to be insufficient to achieve a steady-state in the investigated tissues for these two drugs (Fig. 2, Additional file 1: Fig. S2). Consequently, a continuous subcutaneous administration of up to 10 d via an ALZET osmotic pump was designed based on PK simulations.

Following the administration of a drug, blood samples were withdrawn from the arterial catheter at designated time points to examine the attainment of steady-state. Plasma was immediately obtained by blood sample centrifugation. After decapitation, the DRG, SN, Br, SC, and SM were collected.

K_p,tissue_ was calculated as (Eq. 1)

1 $${K}_{p,tissue}= \frac{{C}_{tot,tissue,ss}}{{C}_{tot,plasma,ss}}$$

where C_tot,tissue,ss_ and C_tot,plasma,ss_ represent the steady-state total concentration in each tissue and plasma, respectively.

The integrity of tissue-to-blood endothelial barriers was assessed in healthy rats after a 5-min IV constant-rate infusion of 400 mg/kg 4 kDa tetramethylrhodamine isothiocyanate (TRIC) dextran, based on the calculated tissue-to-plasma concentration ratio.

### In vitro brain slice assay to evaluate drug brain tissue uptake and binding

The binding and cellular uptake of drugs in the brain was assessed using a brain slice assay based on previously published protocols [29, 30], by estimating the unbound volume of distribution of drug in the brain (V_u,brain_, mL/g brain). Briefly, six 300 μm coronal slices were incubated in 15 mL of artificial extracellular fluid (aECF, pH 7.6) containing the drugs of interest using a cassette approach with up to 5 drugs incubated simultaneously, at 37 °C for 5 h, on an orbital shaker with a rotation speed of 45 rpm, and a constant oxygen supply. At the end of the incubation, buffer and brain slices were sampled and bioanalyzed using UPLC-MS/MS. V_u,brain_ was calculated as the ratio of the drug amount in the brain slice to the measured buffer concentration at the end of the incubation. A V_u,brain_ of 1.0 mL/g brain indicates distribution of unbound drug predominantly in the brain fluid.

### In vitro assay to estimate drug DRG and SN uptake and binding

The unbound volume of drug distribution in the DRG (V_u,DRG_) and SN (V_u,SN_) reflects the drug uptake and binding in the DRG and SN parenchyma. It is crucial that the cellular membrane integrity is preserved during the measurements. To achieve this, a novel approach was used, adapting the brain slice assay to the significantly smaller-sized neural tissues DRG and SN. The method featured two types of incubations: (i) incubation of the DRG and SN collected from drug-naïve rats, in a mixture of drugs in aECF; and (ii) incubation of the DRG and SN collected from rats that received the drug in vivo, in blank aECF. The incubations were performed for 5 h at 37 °C in a 24-well plate, on an orbital shaker with a rotation speed of 100 rpm, and with a constant oxygen supply. At the end of the incubation, the buffer and tissues were sampled and bioanalyzed using UPLC-MS/MS. Assuming that the drug concentration in the buffer at equilibrium represents the interstitial fluid concentration in the DRG or SN, V_u,DRG_ and V_u,SN_ were calculated as the ratio of the drug amount in the DRG or SN, accordingly, to the measured buffer concentration at the end of the incubation.

### In vitro equilibrium dialysis to assess drug plasma protein and tissue binding

The fraction of unbound drug in the plasma (undiluted), and neural and skeletal muscle tissue homogenates (1:9, w:v with PBS, 7.4) was assessed by equilibrium dialysis. Briefly, 100 μL of undiluted plasma or tissue homogenate spiked with a drug of interest was dialyzed against an equal volume of blank PBS for 4–6 h at 37 °C, with shaking at 200 rpm, in a Teflon 96-well equilibrium dialysis device (HTDialysis LLC, Gales Ferry, CT, USA). At the end of incubation, the plasma or tissue, and buffer were sampled and bioanalyzed using UPLC-MS/MS. The fractions of unbound drug in the plasma (f_u,plasma_) and tissue homogenates (f_u,tissue_) were calculated as buffer-to-plasma or buffer-to-tissue concentration ratios, respectively, corrected for the tissue dilution factor [31]. A fraction of unbound drug of 1.0 reflects no binding.

### Bioanalysis

All drugs and their deuterated analogs (internal standards) in the samples, and the respective blanks, standards, and quality controls were bioanalyzed using UPLC-MS/MS. Chromatographic separation was achieved using an ACQUITY UPLC system and MS/MS detection was performed by multiple reaction monitoring using a Xevo TQ-S Micro triple quadrupole mass spectrometer equipped with an electrospray ionization source (Waters Corporation, Milford, MA, USA). The details of sample preparation procedures and UPLC-MS/MS conditions are shown in Additional file 2: Tables S5–S7.

### PK parameters for mapping the extent of transport of unbound drug across endothelial and parenchymal cellular barriers

To assess the extent of drug transport at the DRG, SN, Br, SC, or SM endothelial barriers, unbound tissue extracellular-to-plasma concentration ratio (K_p,uu_) was calculated according to Eq. 2 [23].

2 $${K}_{p,uu}= \frac{{K}_{p,tissue}}{{V}_{u,tissue}\times {f}_{u,plasma}}$$

where K_p,tissue_ is the total tissue-to-plasma concentration ratio at steady-state; f_u,plasma_ is the unbound fraction of drug in the plasma measured by ED; and V_u,tissue_ is the unbound volume of distribution of drug in the respective tissue.

To evaluate the extent of drug transport at the DRG, SN, and Br parenchymal cellular barriers, unbound intracellular-to-extracellular concentration ratio (K_p,uu,cell_) was calculated using Eq. 3 [32]:

3 $${K}_{p,uu,cell}= {V}_{u,tissue}\times {f}_{u,tissue}$$

For interpretation of K_p,uu_ and K_p,uu,cell_ values, see Fig. 1C.

### Statistical analysis

Statistical analyses were performed using GraphPad Prism 9.5.0 for Windows (GraphPad Software, San Diego, CA, USA). For each drug, the differences between the parameters K_p,uu,tissue_, K_p,uu,cell,tissue_, V_u,tissue_, and f_u,tissue_ for each tissue were evaluated by one-way ANOVA followed by Tukey’s or Dunn’s multiple comparison test, as indicated. The significance threshold was set at *p* < 0.05. Data are expressed as the mean ± SD.

The SD for K_p,uu_ and K_p,uu,cell_ was calculated following the law of propagation of error since they were derived from three (Eq. 2) or two (Eq. 3) parameters with uncertainty around the mean of each parameter [33, 34]. Propagation of uncertainty was estimated for both product and quotient of two variables, A and B, using the following equations.

Propagation of uncertainty of K_p,uu,cell_ was calculated according to the product rule. Let A and B be variables with respective SD σ_A_ and σ_B_ and set

4 $$f= A\cdot B$$

Propagation uncertainty for a product, i.e., the SD of *f*, was then calculated as follows:

5 $${\sigma }_{f}\approx \left|f\right|\times \sqrt{{\left(\frac{{\sigma }_{A}}{A}\right)}^{2}+{\left(\frac{{\sigma }_{B}}{B}\right)}^{2}+2\frac{{\sigma }_{AB}}{AB}}$$

The covariance σ_AB_ was calculated with the correlation r as σ_AB_ = rσ_A_σ_B_.

Propagation of uncertainty of K_p,uu_ was calculated according to the quotient rule. Let A and B be variables with respective SD σ_A_ and σ_B_ and set

6 $$f= \frac{A}{B}$$

Propagation uncertainty for a quotient, i.e., the SD of f, was then calculated as follows:

7 $${\sigma }_{f}\approx \left|f\right|\times \sqrt{{\left(\frac{{\sigma }_{A}}{A}\right)}^{2}+{\left(\frac{{\sigma }_{B}}{B}\right)}^{2}-2\frac{{\sigma }_{AB}}{AB}}$$

As abovementioned, the covariance was calculated as σ_AB_ = rσ_A_σ_B_. Considering the innate correlation between the variables, |r|= 0.5 was assumed in all formulas. A negative correlation, i.e., r =  − 0.5, is present between V_u,tissue_ and f_u,plasma_, and f_u,tissue_ while all other parameters were positively correlated, i.e., r = 0.5.

## Results

### Overall considerations of the experimental setup of the study

In the current study, the paclitaxel and vincristine measurements are based on long-term administration using ALZET pumps, while those of other drugs are based on drug administration via 4-h IV infusion. That is because, the steady-state in the blood and in the investigated tissues, prerequisite for an accurate assessment of K_p,tissue_, could not be achieved after 4-h IV infusion for paclitaxel and vincristine (Additional file 1: Fig. S2). Indeed, for paclitaxel, K_p_ values in the five tissues were on average 4–21 times higher following a 240-h constant subcutaneous administration via ALZET pump than those obtained after the 4-h IV infusion, confirming slow attainment of equilibrium in the tissues (Additional file 1: Fig. S2). Because of the limitations associated with paclitaxel formulation for the ALZET pump, the achieved steady-state mean total plasma concentration (C_tot,plasma,ss_) was 12 ng/mL, ca. 18-fold lower than that from the 4-h IV infusion study (Additional file 1: Fig. S1B). We also performed a low-dose IV infusion study, aiming to achieve a similar C_tot,plasma,ss_, to exclude the occurrence of potential concentration-dependent non-linear processes. Indeed, a mean C_tot,plasma,ss_ of 5.3 ng/mL was achieved and K_p_ values in all the tissues (NB; SC was not investigated) were similar to those obtained after the high-dose 4-h IV infusion (Additional file 1: Fig. S2A, Additional file 2: Table S3). The latter rules out the occurrence of any concentration-dependent process in the investigated plasma concentration range and supports the phenomenon of slow equilibration on the tissue side. We observed a similar pattern for vincristine, although because of its severe toxicity, it was only administered for up to 48 h. Vincristine K_p_ values in the Br and SC were 1.7–2.9 times significantly higher following the SC administration than those after 4-h IV infusion (Additional file 1: Fig. S2B, Additional file 2: Table S3).

### Total drug distribution to CIPN sites is drug- and site-dependent

Total drug partitioning across biological barriers in vivo is complex and reflects not only the transport across the endothelial barrier but also binding to plasma proteins, and binding to and uptake by the tissue parenchymal cells. Before investigating unbound drug PK, for context for the ensuing unbound drug PK investigations, we determined total drug distribution (tissue-to-plasma concentration ratios, K_p,tissue_) to different CIPN sites (Fig. 2, Additional file 1: Fig. S2, and Additional file 2: Tables S4 and S8).

Because of the structural and functional differences of neurovascular and muscle microvascular units at the selected CIPN-specific anatomical sites (Fig. 1), we also assessed the paracellular transport in healthy rats, using 4 kDa TRITC-dextran (Fig. 2L). We detected the lowest tissue-to-serum concentration ratio in the brain (0.0012, i.e., 0.12%), indicating low paracellular transport (Fig. 2L). Although the tissue-to-serum concentration ratios at the BNB, BDB, and BSMI were substantially higher than that at the BBB, the paracellular transport across these barriers remained restricted, as the mean values did not exceed 0.05 (5%). The total tissue-to-serum concentration ratios of TRITC-dextran revealed the following rank order: DRG = SN > SM >  > SC > Br, directly reflecting the barrier tightness: BBB > BSCB >  > BSMI > BNB = BDB. Hence, this confirmed that the contribution of paracellular transport to the extent of transport across the blood-to-tissue barriers is low.

### Unbound drug transport at the blood–tissue barriers at CIPN sites is drug- and site-dependent

**Fig. 3:**
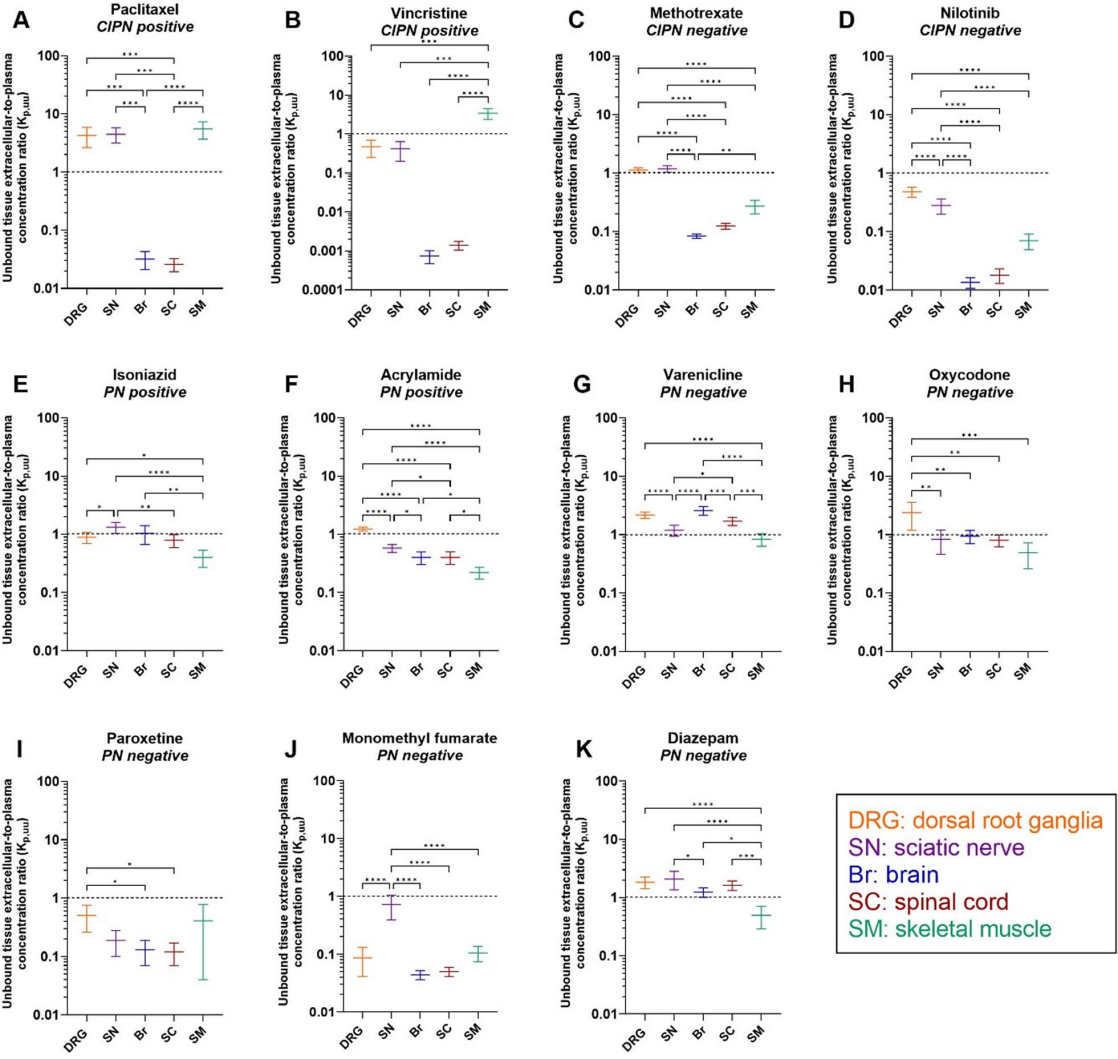
Scatter plots of unbound tissue extracellular-to-plasma concentration ratio (K_p,uu_) at steady-state for the indicated drugs in the specified tissues. This parameter describes the extent of unbound drug transport across the BDB (DRG), BNB (SN), BBB (Br), BSCB (SC), and BSMI (SM), accordingly. **A–K** Measurements for the different drugs, with their CIPN and PN propensity indicated. Black dashed line, K_p,uu_ = 1, indicates predominant passive diffusion or mutually compensated influx and efflux; K_p,uu_ < 1 indicates predominant active efflux, while K_p,uu_ > 1 indicates predominant active uptake. Data are presented as the mean ± SD estimated using the error propagation method [51]. *p < 0.05, **p < 0.01, ***p < 0.001, or ****p < 0.0001 (ordinary one-way ANOVA with Tukey’s multiple comparison test). NB: note the differences in scale

We then using Eq. 2 calculated K_p,uu,tissue_ in order to determine the extent of unbound drug transport across endothelial barriers (Fig. 3). Paclitaxel, vincristine, methotrexate, and nilotinib were efficiently effluxed at the BBB and BSCB, as reflected by K_p,uu,Br_ and K_p,uu,SC_ ranging from 0.00074 to 0.12 (Fig. 3A–D, Additional file 1: Fig. S3, and Additional file 2: Table S8). The largest parameter difference for paclitaxel was 214-fold (*p* < 0.0001) between K_p,uu,SM_ and K_p,uu,SC_; while the most prominent difference for vincristine was 4600-fold (*p* < 0.0001) between K_p,uu,SM_ and K_p,uu,Br_ (Fig. 3A and B). Remarkably, K_p,uu,DRG_, K_p,uu,SN_, and K_p,uu,SM_ for paclitaxel indicated extensive active influx at the BDB, BNB, and BSMI, respectively (Fig. 3A). We also observed an active uptake of vincristine at the BSMI (Fig. 3B). Despite a substantially higher K_p,uu_ to the PNS than that to the CNS, active efflux dominated the transport of vincristine and nilotinib at the BDB and BNB (Fig. 3B and D). The active efflux of nilotinib at the BSMI was, however, as efficient as that at the CNS endothelial barriers, with no significant differences between K_p,uu,SM_ and K_p,uu,Br_, K_p,uu,SC_ (Fig. 3D). K_p,uu,DRG_ and K_p,uu,SN_ for methotrexate were close to unity, indicating passive diffusion, or mutually compensated influx and efflux at the PNS endothelial barriers (Fig. 3C). Nevertheless, K_p,uu,SM_ for methotrexate was below unity, despite being significantly higher than K_p,uu,Br_. The striking difference in the behavior of CIPN-positive drugs was an active uptake at BSMI not present for CIPN-negative drugs.

For the PN-positive drugs, isoniazid transport across the PNS and CNS endothelial barriers was dominated by either passive diffusion or mutually compensated influx and efflux, with K_p,uu_ close to 1 (Fig. 3E). However, active efflux dominated the transport of isoniazid across the BSMI (Fig. 3E). By contrast, acrylamide exhibited active efflux at all interfaces except the BDB, with K_p,uu,DRG_ close to 1 (Fig. 3F).

For the PN-negative drugs, varenicline and oxycodone both showed predominant active uptake at the BDB but not at the BNB (Fig. 3G and H). We also observed active influx of varenicline at both CNS barriers (Fig. 3G). Further, paroxetine was actively effluxed at all endothelial barriers (Fig. 3I). Similarly, monomethyl fumarate demonstrated predominant efflux at all interfaces, with K_p,uu_ ≤ 0.1, except at the BNB (Fig. 3J). Finally, we observed a potential active influx of diazepam at the BNB (Fig. 3K).

Taken together, characteristics of transport at the blood–tissue barriers for CIPN-positive/negative drugs and PN-positive/negative drugs were drug and tissue specific.

### Different drugs exhibit different plasma protein binding and intra-tissue drug distribution at CIPN sites

**Fig. 4:**
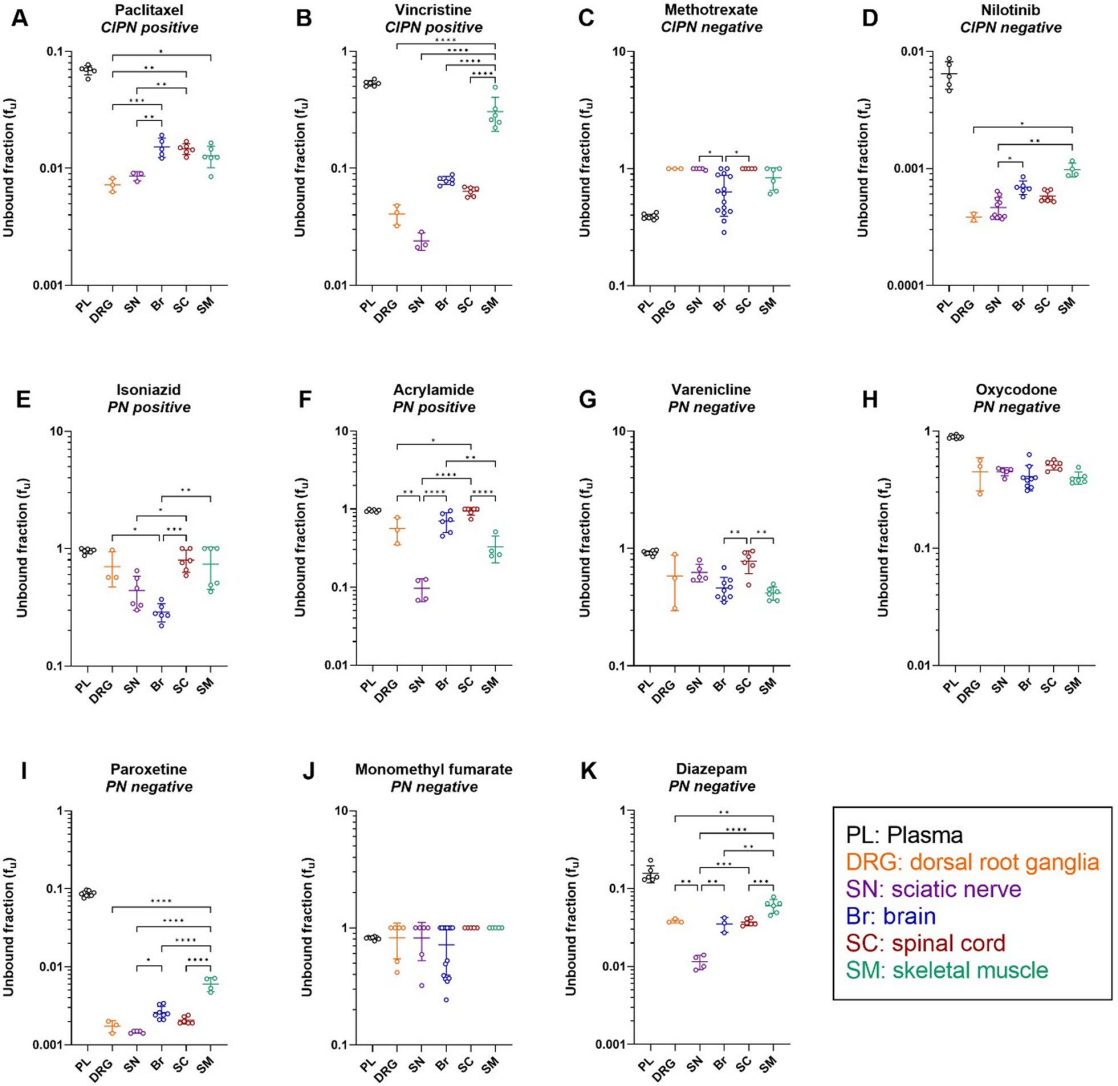
Scatter dot plots of unbound fraction (f_u_) of the indicated drugs in the plasma in the specified tissues. This parameter describes the extent of drug binding to plasma proteins, as well as DRG, SN, Br, SC, and SM tissue constituents. **A–K** Measurements for the different drugs, with their CIPN and PN status indicated. Data are presented as the mean ± SD. See Additional file 2: Table S4 for the details of the number of biological and technical replicates of each experiment. *p < 0.05, **p < 0.01, ***p < 0.001, or ****p < 0.0001 (ordinary one-way ANOVA with Tukey’s multiple comparison test or Kailas–Kruskal test followed by Dunn’s multiple comparison test). NB: note the differences in scale

We next determined the binding of the selected drugs to plasma proteins and tissue constituents. The former varied dramatically, with f_u,plasma_ ranging from 0.0065 for nilotinib (extremely high binding) to 0.96 for acrylamide (almost no binding) (Fig. 4 and Additional file 2: Table S3). The tissue binding of these drugs also varied dramatically, with the lowest f_u,tissue_ of 0.00038 for nilotinib in the DRG, and the highest f_u,tissue_ of 1.0 for methotrexate in the DRG and SC, and monomethyl fumarate in the SC and SM (Additional file 2: Table S4). Further, we found significant inter-tissue differences in f_u,tissue_ for all the investigated drugs, except for oxycodone and monomethyl fumarate (Fig. 4 and Additional file 2: Table S4). Correlation matrix analysis revealed that the SC and DRG binding properties of the drugs were highly correlated with brain tissue binding, with a correlation coefficient (r) of 0.96 and 0.90, respectively (Additional file 1: Fig. S4).

**Fig. 5:**
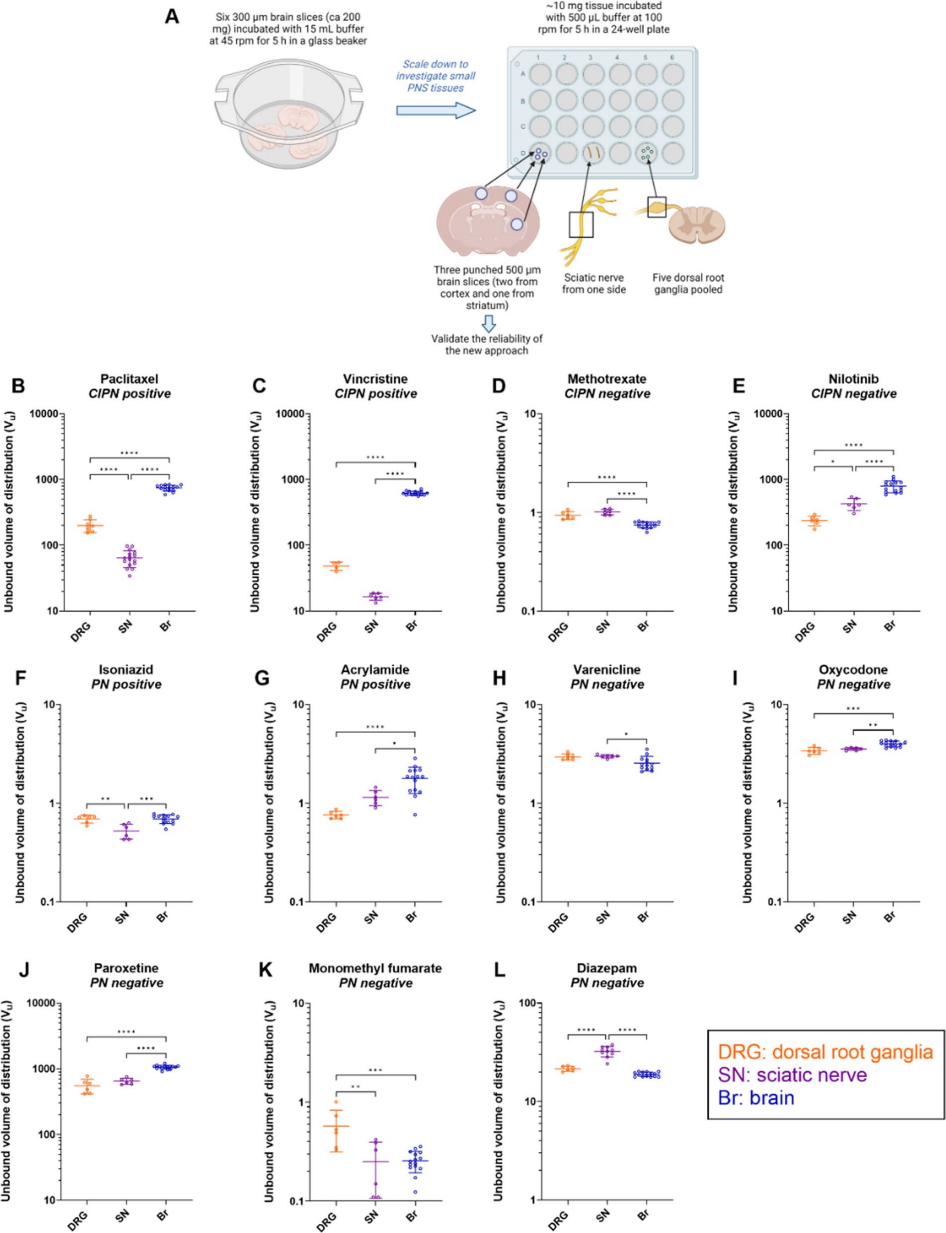
Measurement of the unbound volume of distribution (V_u_) of the indicated drugs in the specified tissues. This parameter describes both the cellular uptake and binding in the DRG, SN, and Br. **A** Experimental overview. **B–L** Scatter dot plots of V_u_ of the different drugs, with their CIPN and NP propensity indicated. Data are presented as the mean ± SD, n = 3–8 biological replicates (1–5 technical replicates per each biological replicate). *p < 0.05, **p < 0.01, ***p < 0.001, or ****p < 0.0001 (ordinary one-way ANOVA with Tukey’s multiple comparison test). NB: note the differences in scale

We then determined the unbound volume of distribution (V_u_), which describes the intra-tissue distribution properties in the DRG, SN, and brain. To do this, we used a novel in vitro PNS tissue distribution method and brain slice assay, accounting for both, intracellular uptake and binding in tissues, while preserving the cellular integrity and pH gradient (Fig. 5 and Additional file 2: Table S4). The uptake/binding of the selected drugs in the DRG, SN, and brain parenchymal cells varied markedly, with the largest V_u_ of 1058 mL/g tissue for paroxetine in the brain, and the smallest V_u_ of 0.25 mL/g tissue for monomethyl fumarate in the SN (Fig. 5 and Additional file 2: Table S4).

### Unbound drug transport across the cellular barriers at CIPN sites is drug- and site-dependent

**Fig. 6:**
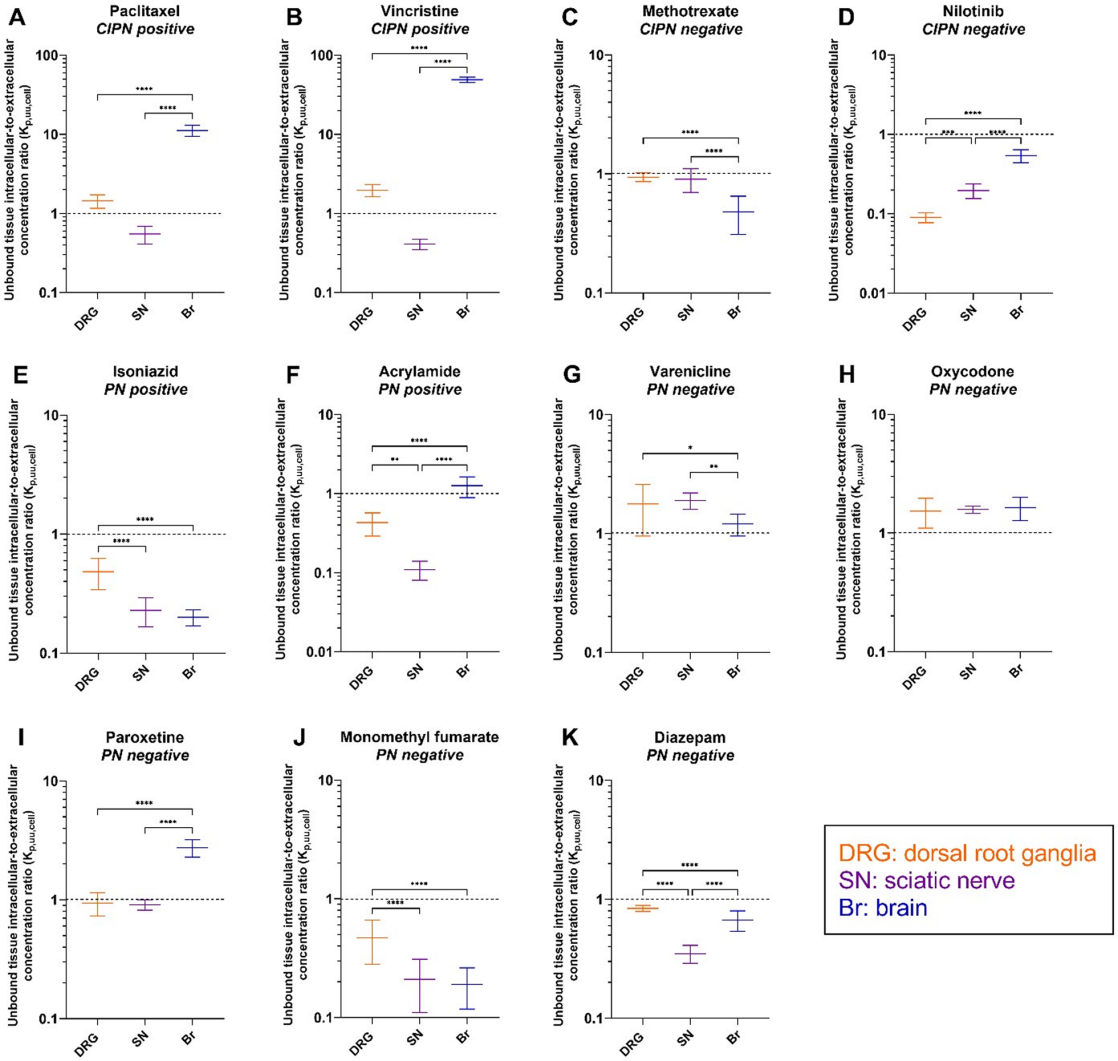
Scatter plots of unbound intracellular-to-extracellular concentration ratio (K_p,uu,cell_) of the indicated drugs at the specified tissues. This parameter describes the extent of unbound drug transport across the cellular barriers in the DRG, SN, and Br parenchyma. **A–K** Measurements for the different drugs, with their CIPN and PN status indicated. Black dashed line, K_p,uu,cell_ = 1, indicates predominant passive diffusion or mutually compensated influx and efflux; K_p,uu,cell_ < 1 indicates limited intracellular entry; K_p,uu,cell_ > 1 indicates predominant accumulation inside the cells. Data are presented as the mean ± SD estimated using the error propagation method [51] . *p < 0.05, **p < 0.01, ***p < 0.001, or ****p < 0.0001 (ordinary one-way ANOVA with Tukey’s multiple comparison test). NB: note the differences in scale

K_p,uu,cell_ estimated using the CMA-CIPN (Eq. 3) describes whether a drug tends to accumulate intracellularly or has limited entry into the cells, here, the DRG, SN and brain (Fig. 6, Additional file 1: Fig. S5, Additional file 2: Table S8).

The two CIPN-positive drugs, paclitaxel and vincristine, substantially accumulated inside the cells in the brain parenchyma, with K_p,uu,cell,Br_ of 11 and 49, respectively. Their accumulation in the DRG parenchymal cells was significantly less pronounced, with K_p,uu,cell,DRG_ less than 2. Of note, the entry of these two drugs into SN parenchymal cells was limited, reflected by K_p,uu,cell,SN_ < 1. Methotrexate and nilotinib, the two CIPN-negative drugs, did not show any active intracellular accumulation in the DRG, SN, or brain, with K_p,uu,cell_ close to or less than 1. Similarly, we found no intracellular accumulation for the two PN-positive drugs isoniazid and acrylamide. Finally, the PN-negative drugs exhibited different cell-partitioning capability: varenicline and oxycodone showed active intracellular accumulation in the three tissues; monomethyl fumarate and diazepam show no active accumulation inside the cells (K_p,uu,cell_ < 1); and paroxetine only accumulated intracellularly in the brain.

### Interplay between Kp,uu and Kp,uu,cell governs CIPN site exposure

To illustrate how drug transport across tissue-specific endothelial and cellular barriers determines the target-site exposure, we performed a simulation of steady-state unbound drug concentrations in the investigated tissue compartments for all drugs, assuming an arbitrary value for a total plasma concentration of 100 ng/mL (Additional file 2: Table S8). It presents the obtained concentration values for representative drugs calculated based on the obtained PK and neuropharmacokinetic parameters.

**Fig. 7:**
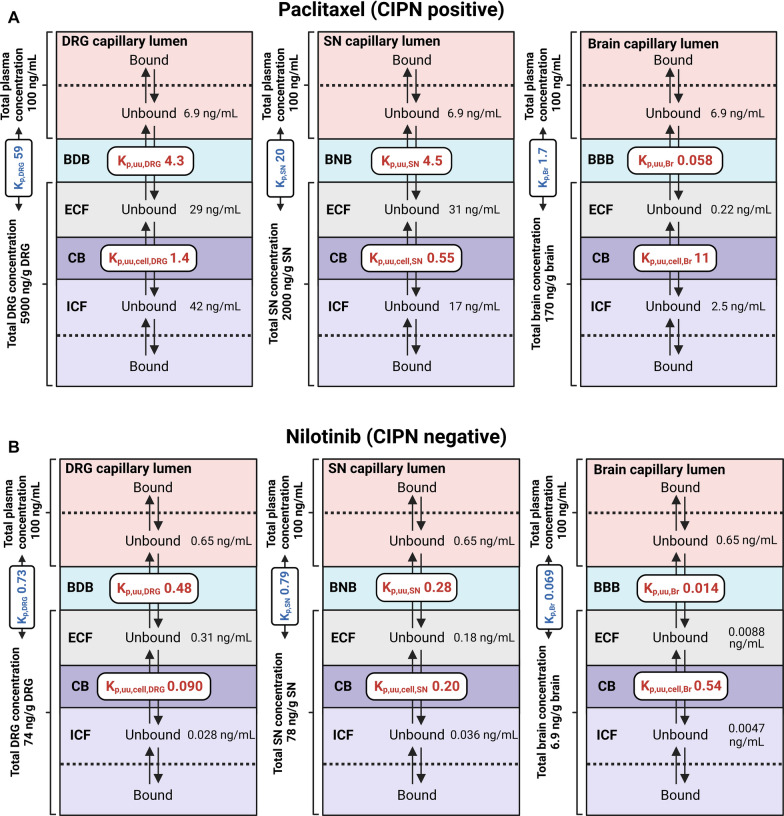

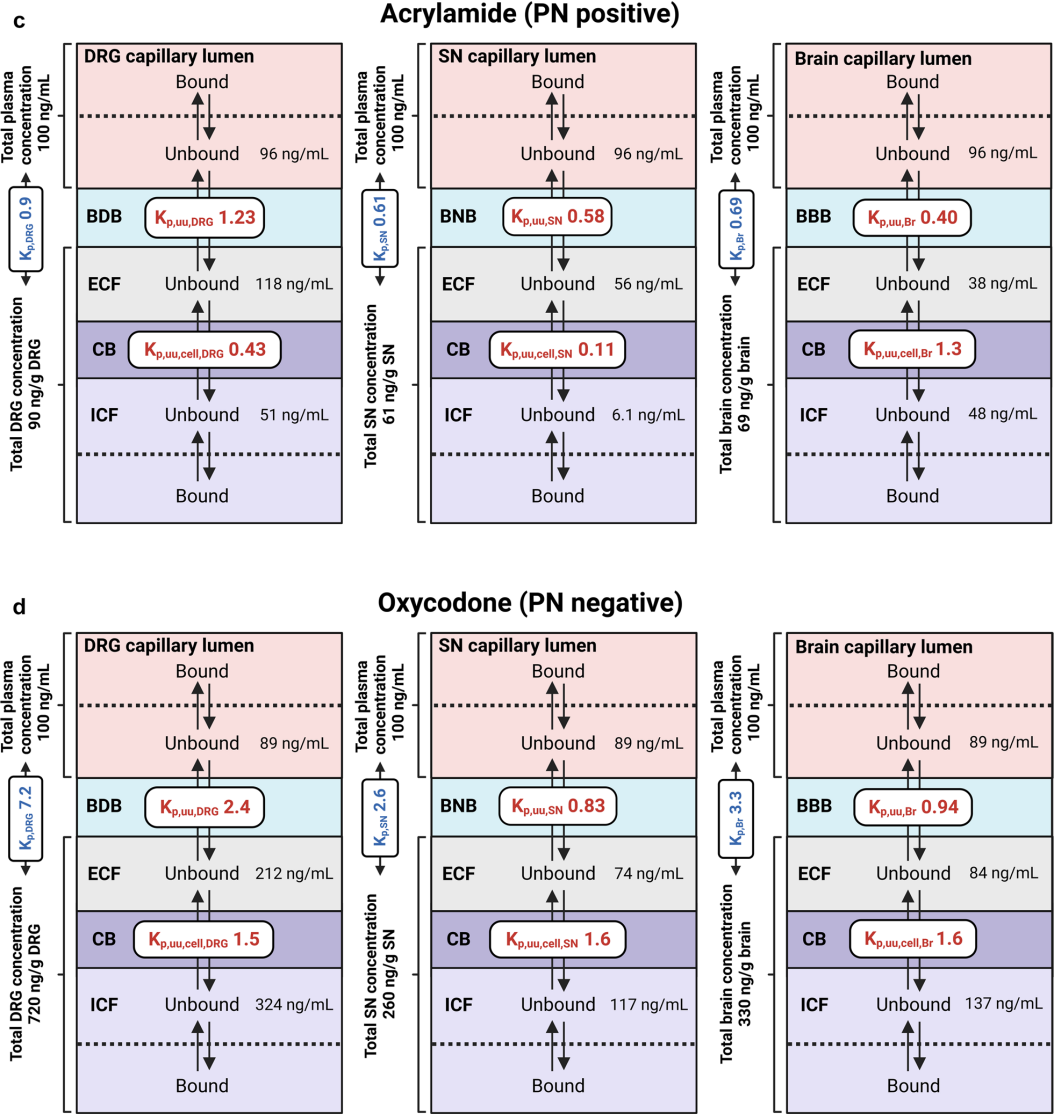
Estimated steady-state unbound concentrations of representative drugs in the plasma, and in the extracellular (ECF) and intracellular (ICF) spaces of the dorsal root ganglia (DRG), sciatic nerve (SN), and brain (Br), with total drug concentrations in the respective tissues indicated. f_u,plasma_, K_p_, K_p,uu_, and K_p,uu,cell_ measured in the current study were used for the calculations, with the assumption of the total plasma concentration of 100 ng/mL for each drug. **A–D** Specific drug data. See Additional file 2: Table S8 for the results of the simulation exercise for all drugs tested in the current study

For paclitaxel (CIPN-positive), the simulated unbound DRG and SN extracellular concentrations were similar, but were 134-fold higher than that in the corresponding brain compartment because of a less efficient efflux at the BDB and BNB (Fig. 7A). Despite more extensive intracellular accumulation in the brain, the unbound paclitaxel intracellular concentration in the brain remained 6.8-fold and 17-fold lower than that in the SN and DRG, respectively. Remarkably, bound paclitaxel accounted for a major portion (99%) of its total exposure. Notably, total paclitaxel concentration in the SN and DRG was 12-fold and 35-fold higher than in the brain, respectively.

For nilotinib (CIPN-negative), the simulated unbound DRG extracellular concentration was the highest among the three tissues because of the least efficient efflux at the BDB, compared with that at the BNB and BBB (Fig. 7B). However, because of limited entry into the DRG parenchymal cells (K_p,uu,cell_ < 0.1), the unbound nilotinib intracellular concentration in the DRG was slightly lower than that in the SN, but still sixfold higher than that in the brain.

For acrylamide (PN-positive), the extracellular concentration in the DRG was 2.1- and 3.1-fold higher than that in the SN and brain, respectively, because of the lack of efflux at the BDB (Fig. 7C). Furthermore, the intracellular exposure in the DRG and brain was approximately eightfold higher than that in the SN because of limited entry into the SN cells.

Finally, oxycodone (PN-negative) reached the highest extracellular concentration in the DRG because of its active uptake at the BDB. The intracellular concentration of oxycodone in the DRG was also higher than that in the brain and SN, as oxycodone tended to accumulate inside the DRG, SN, and brain parenchymal cells to a similar extent.

## Discussion

In the current study, we investigated the role of drug transport at biological barriers to target-site exposure in the context of CIPN. This is the first systematic evaluation of the transport of small-molecule drugs across both, the endothelial and parenchymal cellular barriers at conventional and non-conventional CIPN sites, focusing on unbound drug disposition. We have leveraged a novel extension of the CMA methodology, CMA-CIPN, to show that the extent of drug transport across the endothelium at the different CIPN sites is markedly diverse, with the transport across the BDB in general more profound than that across other biological barriers. Further, the intracellular distribution in tissue parenchyma was markedly different between the CIPN sites for the drugs studied. Remarkably, the investigated CIPN-positive drugs, but not the investigated CIPN-negative drugs, exhibited intracellular accumulation. These findings collectively represent a useful resource for the generation of novel preventive and treatment approaches for CIPN.

Evaluation of the extent of transport across the endothelial barriers at CIPN sites revealed that for both, CIPN-positive and -negative drugs, transport at conventional sites across the BDB and BNB was significantly greater than that across the BBB and BSCB (Fig. 3A–D). The differences in K_p,uu_ of the two CIPN-positive drugs paclitaxel and vincristine between PNS and CNS tissues were substantial, which might explain why they induce neurotoxicity only in the PNS but not in the CNS. This almost completely rules out the contribution of CNS exposure to CIPN development for paclitaxel and vincristine, yet the role of CNS in CIPN development by other CIPN-D needs additional investigations. Intriguingly, paclitaxel exhibited K_p,uu,DRG_ and K_p,uu,SN_ of approximately 4.4 (Fig. 3A), suggesting active influx at the BDB and BNB. Active uptake at the BDB might potentially be mediated by Oatp1b2 (OATP1B1 and 1B3 in human), an influx transporter that mediates paclitaxel-induced PN [17]. The precise location of OATP1B1 (DRG neuronal cell membrane vs. the BDB) is yet to be determined [16]. Detailed transcriptomic and/or proteomic analyses of isolated DRG microvasculature and parenchymal tissue are warranted to determine the localization of Oatp1b2 and other, relevant transporters in the DRG.

It is worth to mention that for selected CIPN-D paclitaxel and vincristine, the PK is strongly linked to their pharmacodynamic mechanism, i.e., interaction with the tubulin. Kuh et al. investigated the intracellular behavior of paclitaxel in human breast MCF7 tumor cells lacking P-glycoprotein, supported by mathematical modeling describing non-linear cellular PK [35]. Concentration-dependent changes in paclitaxel PK were associated with the saturation of the binding sites at 1000 nM and higher extracellular concentrations and paclitaxel-induced increases in total tubulin [35]. The latter, is highlighting the complexity of the intracellular PK of paclitaxel governed by potential active transport mechanisms occurring on the level of cellular membrane as well as specific and non-specific binding.

Experimental features not fully explored in the CMA-CIPN methodology that may impact the assessment of exposure of drugs in the CIPN-sites, as well as the extent of the unbound drug tissue-to-blood concentration ratios, include estimation of both the residual blood and the extent of paracellular transport in CIPN-sites. The residual blood in the tissue samples depends on the method of sacrification of the animal. The latter has been extensively studied in the field of CNS drug delivery with the proposal of a drug-specific correction for brain residual volume by Fridén et al. [36]. Remaining in the tissue blood may contribute to the overestimation of the drug concentration in the tissue when the drug has a very low total brain-to-plasma concentration ratio and plasma protein binding that exceeds drug brain tissue binding. This type of combination among investigated drugs could be seen for methotrexate and monomethyl fumarate (Additional file 2: Tables S4, S8). The impact could be more pronounced for the brain and spinal cord as in both cases total tissue-to-plasma concentration ratio is below 0.05. After applying correction according to a simplified method by Fridén et al. K_p,brain_ of methotrexate changed from 0.02 to 0.015 and K_p,brain_ of monomethyl fumarate changed from 0.009 to almost 0. Overall, this does not affect the conclusion regarding the extent of the BBB transport, however, it highlights the importance of the correction for the residual blood volume. The development of a drug-specific correction method for CIPN-sites will improve the accuracy of the proposed CMA-CIPN method for a set of drugs characterized by a very low exposure in the CIPN tissues and is now under development by the authors. In addition, the contributive role of paracellular transport to the net flux across blood-to-tissue barriers at CIPN-site could be underestimated in this study. We have used intravenous constant rate infusion of 4 kDa TRITC-dextran for the estimation of paracellular transport (Fig. 2L) which has shown that paracellular transport is ca 5% in DRG and SN. However, the usage of radiolabeled sucrose could better characterize the paracellular transport for small molecular weight drugs.

The investigated CIPN-negative drugs showed either active efflux (nilotinib) or passive diffusion/mutually compensated efflux and influx (methotrexate) at the BDB and BNB. The PK properties of methotrexate predispose it to high unbound exposure at CIPN sites, in particular in the DRG. This may explain why in some clinical cases, e.g., treatment of rheumatoid arthritis, methotrexate administration is associated with the development of PN [37]. However, additional studies should be performed to verify this conclusion.

Strikingly, unlike CIPN-negative drugs, the two CIPN-positive drugs tested exhibited active uptake across the BSMI. For all the other investigated drugs, K_p,uu,SM_ ≤ 1. Although chemotherapy-induced muscle effects are well documented, the molecular mechanisms are unclear [8, 12]. We here showed pronounced uptake of paclitaxel and vincristine into SM across the endothelial interface, possibly linking high SM exposure and CIPN development ((Additional file 1:Fig. S6). Unlike the CNS and PNS barriers, the BSMI is usually considered not to be a true barrier for drugs, despite very little quantitative evidence to support that. Morphologically, the muscle microvascular unit consists of endothelial cells and pericytes supported by basal membrane (Fig. 1A), with functionally heterogeneous extrafusal and intrafusal capillaries [38]. Of note, while muscle endothelial cells express tight junction proteins similar to the BBB and BNB, and some drug transporters [39], their vesicular transport is more pronounced than that at the nervous system barriers [38]. The drug concentration asymmetry across the BSMI observed herein indicates that drug transport into the SM is governed by both, active influx and efflux, and should not be assumed to be a passive process. Indeed, recent advances in the understanding of the molecular makeup of endothelial cells of the endomysium and accumulating evidence on the potential differences in molecular transport therein support it being a potential barrier for drugs [39–42]. Our findings are also in line with a recent microdialysis study where predominant efflux or influx transport across the SM endothelial barrier (K_p,uu,SM_ < 1 or > 1) was also found for 14 marketed drugs [42]. Similar findings were also reported for 56 tested compounds where 15 out of 56 compounds showed rather limited penetration at BSMI with K_p,uu,SM_ below 0.3 and also 2 out of 56 compounds demonstrated active uptake with K_p,uu,SM_ higher than 2 [43]. Delineation of the contributive role of SM exposure in the development of CIPN will require investigation of other CIPN-Ds, including characterization of transport across extrafusal and intrafusal capillaries.

We found that the extent of drug transport across parenchymal cellular barriers at CIPN sites was highly variable, and drug- and tissue-dependent. Remarkably, paclitaxel and vincristine showed extremely high accumulation in brain parenchymal cells (K_p,uu,cell,Br_ 11 and 49, respectively), relatively lower intracellular accumulation in the DRG (K_p,uu,cell,DRG_ 1.4 and 2.0, respectively), and limited intracellular entry in the SN (K_p,uu,SN_ 0.55 and 0.41, respectively). This distinct intracellular distribution may be attributed to the number of neurons and the different expression levels of β-tubulin (an intracellular protein that binds paclitaxel and vincristine) between the DRG, SN, and brain [44, 45]. Importantly, a combination of the pronounced transport across endothelial and parenchymal barriers at PNS sites would lead to high intracellular exposure of both unbound and bound paclitaxel and vincristine (Additional file 1: Fig. S6, Additional file 2: Table S8). The extent of cellular barrier transport for the other investigated drugs was less variable between the tissues, reflecting a potential similarity between their transport mechanisms.

Further, the PN-positive drugs displayed less dramatic differences between CIPN sites than CIPN-positive drugs, with K_p,uu_ values slightly above 1 at the BNB for isoniazid and at the BDB for acrylamide. As both drugs are highly unbound in the plasma, the overall unbound extracellular drug exposure at the respective sites could be high, depending on the systemic exposure. Overall, we did not identify any commonalities between the PK properties of the investigated CIPN- and PN-positive drugs, except for the high extent of transport across the BDB/BNB.

PN is a debilitating and painful condition, with no preventive or curative treatment available. The data obtained in the current study indicate that the PK at CIPN site may explain why certain symptomatic treatment approaches are more efficient than others. For instance, oxycodone and varenicline exhibited high unbound exposure at CIPN sites. Active uptake at the BDB is likely therapeutically beneficial, contributing to the clinical efficacy of oxycodone in alleviating neuropathic pain, with a longer-lasting analgesic effect than that of morphine [46, 47], as well as an analgesic effect of varenicline [24]. Further, from a potential CIPN treatment point of view, it is worth mentioning that the main active metabolite of dimethyl fumarate, monomethyl fumarate, is considered to be membrane-impermeable [48]. The latter is indeed supported by our findings of the relatively low K_p,uu,tissue_ and K_p,uu,cell_ values for monomethyl fumarate in all tissues, except for the SN, wherein K_p,uu,SN_ is close to 1 (Fig. 3J). Thus, this tissue-specificity of monomethyl fumarate may explain the observed preventive effect of dimethyl fumarate, which is rapidly metabolized to monomethyl fumarate, in the management of oxaliplatin-induced PN [49]. In addition, the extent of paroxetine transport across endothelial and cellular barriers at CIPN sites was relatively low, which may underly its ineffectiveness in the treatment of PN [25]. In the future, CMA-CIPN should be used to investigate the behavior of the antidepressant duloxetine, currently the most effective treatment for CIPN, to unravel the PK contribution to in-class differences in treatment efficiencies. It is important to bear in mind that the PK of tested drugs could be affected by pathological conditions, including CIPN.

In order to determine K_p,uu_ and K_p,uu,cell_, it is imperative to reliably estimate the relationship between total and unbound extracellular concentrations in in vivo-like settings and determine the unbound volume of distribution in the tissue. In the current study, we developed a novel PNS tissue incubation assay that enables, for the first time, the evaluation of drug disposition in the PNS in a similar manner as is performed for the CNS. However, as a limitation of this approach, unlike the brain slice assay, the obtained V_u,DRG_ and V_u,SN_ values cannot be validated using microdialysis, i.e., the “gold standard” method, because of tissue size limitations. From a methodological standpoint, tissue homogenates with equilibrium dialysis could be used to characterize the relationship between total and unbound drug fraction in the tissues [50]. However, there are inherent differences between the investigation of binding in tissue homogenates (f_u,tissue_) and tissue pieces with preserved cellular integrity (V_u,tissue_) [23, 51]. Herein, we used V_u,tissue_ to estimate both K_p,uu_ and K_p,uu,cell,_ which offers a clear methodological advantage. Of note, previous tissue homogenate-based assessments of K_p,uu_ values revealed the following rank order for the investigated drugs: DRG > SN > SC > brain [50]. By contrast, we observed that pattern for only four of the drugs tested herein, indicating individual drug-specific distribution at CIPN sites (Fig. 3). Investigation of additional drugs representing diverse pharmacological classes and physicochemical space using CMA-CIPN will help to resolve this methodological conundrum.

## Conclusions

In conclusion, in the current study, we demonstrated critical features of (unbound) drug disposition into the PNS and SM, and its differences from drug disposition into the CNS. The obtained PK profiles of CIPN-D in rat model, which recapitulate the relationship between plasma and CIPN site drug exposure, can be used as inputs for mathematical modeling to predict the target-site exposure in human, e.g., using an allometric scaling approach to simulate various “what if” scenarios, to inform new strategies on how to monitor plasma exposure in patients during chemotherapy. Ultimately, this will lower the risk of CIPN, and also aid the development of novel translatable approaches to prevent or mitigate CIPN.

### Supplementary Information

**Additional file 1.  **Supplementary Figures S1-S6 | S1-S6 figures with figure legends.

**Additional file 2.** Supplementary Tables S1-S7 | S1-S7 tables with titles.

**Additional file 3.** Supplementary Materials and Methods section | Extended Materials and Methods section.

## Data Availability

The datasets generated in the current study and/or analyzed during the current study are available from the corresponding author on reasonable request.

## Abbreviations

CIPN: Chemotherapy-induced peripheral neuropathy
PN: Peripheral neuropathy
CIPN-causing drugs: CIPN-D
PNS: Peripheral nervous system
CNS: Central nervous system
DRG: Dorsal root ganglia
SN: Sciatic nerve
Br: Brain
SC: Spinal cord
SM: Skeletal muscle
BDB: Blood–dorsal root ganglion barrier
BNB: Blood–nerve barrier
BBB: Blood–brain barrier
BSCB: Blood–spinal cord barrier
BSMI: Blood–skeletal muscle interface/barrier
CB: Cellular barrier
CMA: Combinatory Mapping Approach
CMA-CIPN: Combinatory Mapping Approach for CIPN
PK: Pharmacokinetics
K_p_: Total tissue-to-plasma concentration ratio
K_p,uu_: Unbound tissue extracellular-to-plasma concentration ratio
K_p,uu,cell_: Unbound tissue intracellular-to-extracellular concentration ratio
V_u,tissue_: Unbound volume of distribution in tissue
f_u,plasma_: Unbound fraction in plasma
f_u,tissue_: Unbound fraction in tissue
