# Supplementary material for: Analysis of the contributing role of drug transport across biological barriers in the development and treatment of chemotherapy-induced peripheral neuropathy
Source: Fluids Barriers CNS. 2024 Feb 8;21:13. doi: 10.1186/s12987-024-00519-7 (PMC10854123; doi:10.1186/s12987-024-00519-7)
Supplement: Supplementary file 1 — Additional file 1. Supplementary Figures S1-S6 | S1-S6 figures with figure legends. [file 12987_2024_519_MOESM1_ESM.docx]

# **Supplementary figures S1-S6 and figure legends**

**Analysis of the Contributing Role of Drug Transport Across Biological Barriers in the Development and Treatment of Chemotherapy-Induced Peripheral Neuropathy**

Yang Hu^1,2^, Milda Girdenyté^1,3^, Lieke Roest^1^, Iida Liukkonen^1^, Maria Siskou^1^, Frida Bällgren^1^, Margareta Hammarlund-Udenaes^1^, and Irena Loryan^1,^*

^1^ Translational Pharmacokinetics-Pharmacodynamics group, tPKPD, Department of Pharmacy, Faculty of Pharmacy, Uppsala University, Box 580, SE-751 23, Uppsala, Sweden

^2^ Current affiliation: Discovery ADME, Drug Discovery Sciences, Boehringer Ingelheim RCV GmbH & Co KG, A-1121, Vienna, Austria

^3^ Pharmacy and Pharmacology Center, Institute of Biomedical Sciences, Faculty of Medicine, Vilnius University, M.K. Čiurlionio, Str. 21/27, LT-03101 Vilnius, Lithuania

* Corresponding author:

Irena Loryan, MD, PhD

Translational PKPD group, Department of Pharmacy

Box 580, 751 23 Uppsala, Sweden


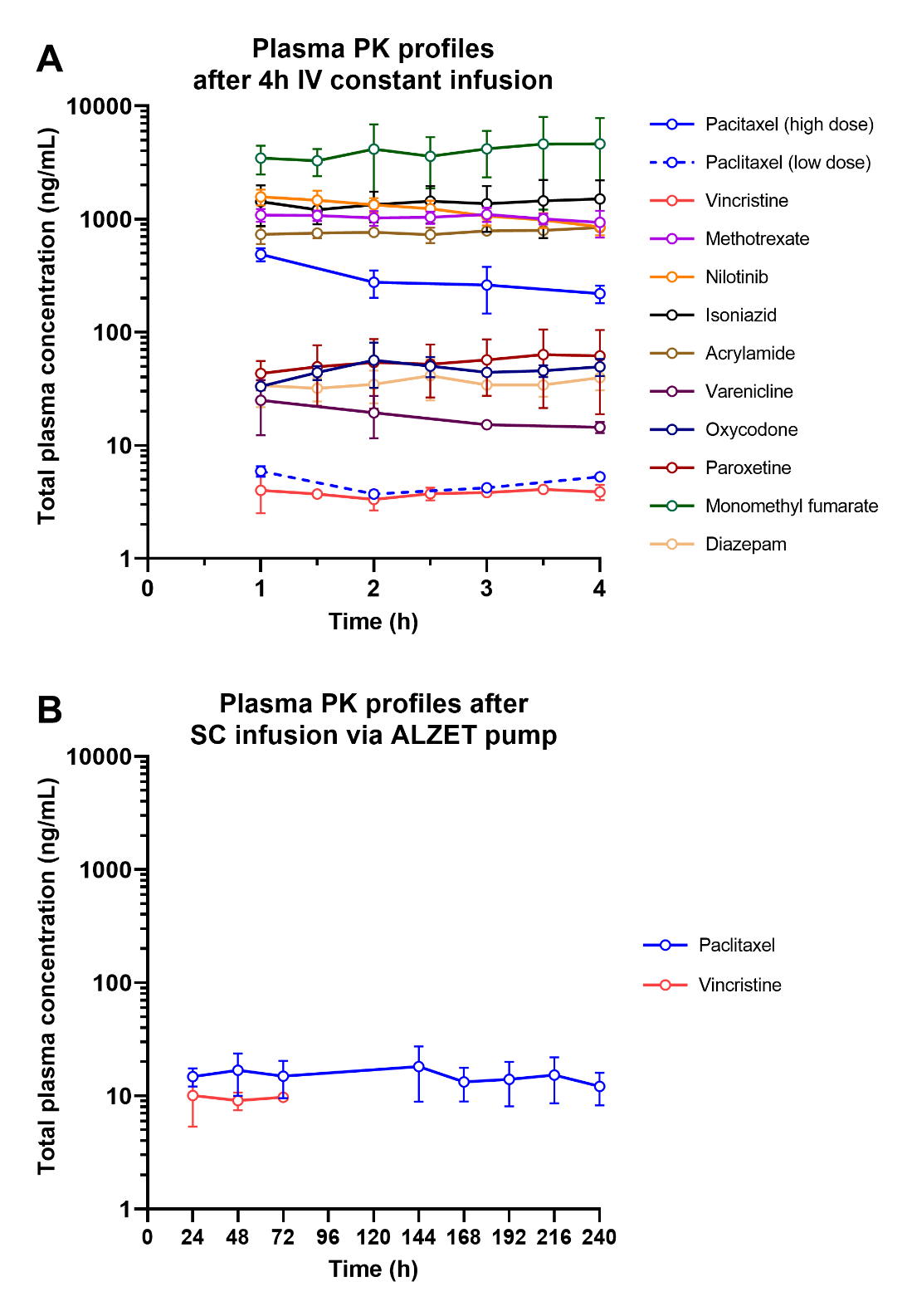


**Figure S1.**

Total plasma concentration-time profiles of investigated drugs following 4-h intravenous constant infusion **(A)** and after subcutaneous infusion of paclitaxel and vincristine via ALZET osmotic pump **(B).**


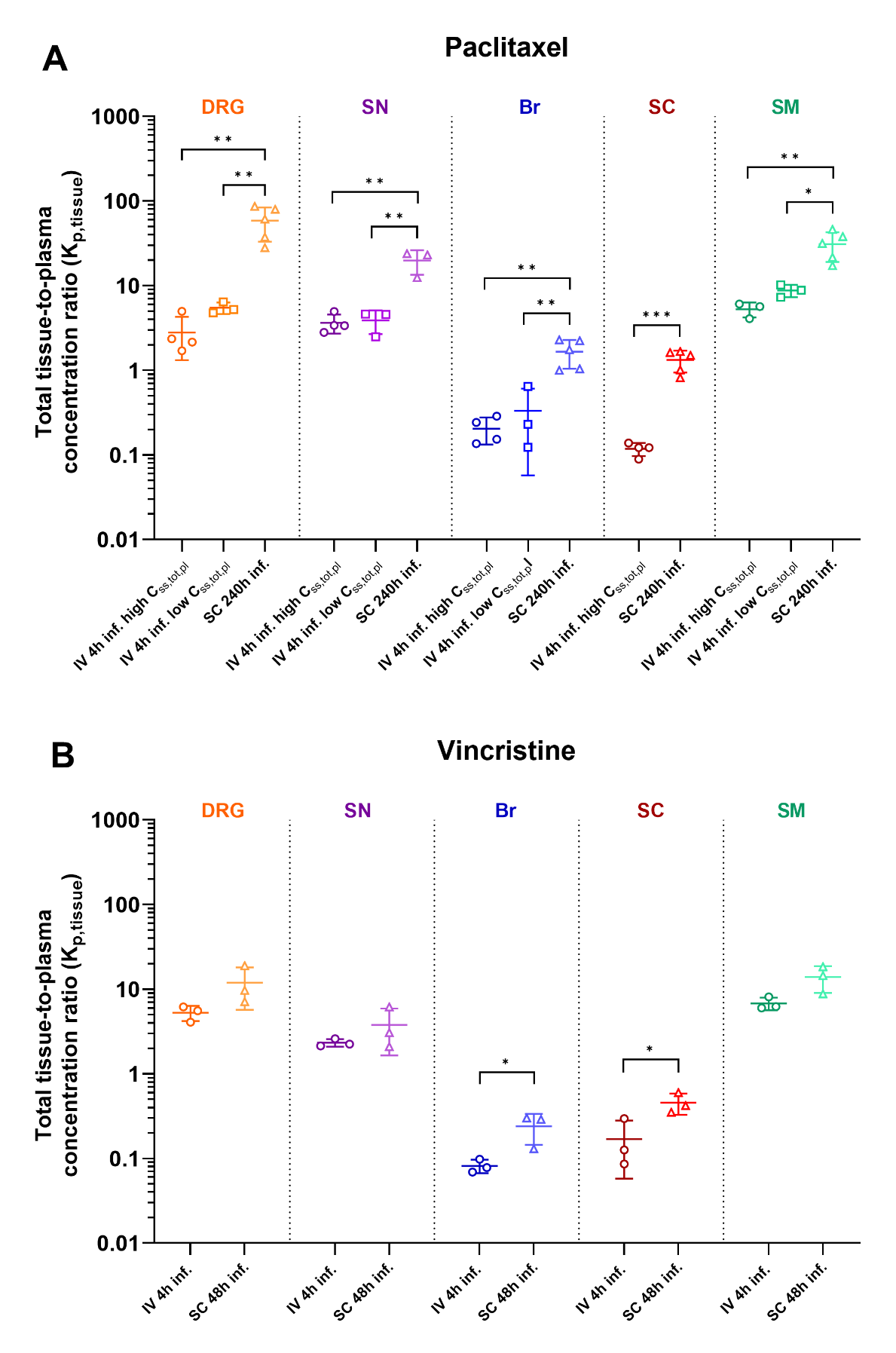


**Figure S2.**

Total tissue-to-plasma concentration ratio (K_p,tissue_) of (**A**) paclitaxel and (**B**) vincristine in dorsal root ganglia (DRG), sciatic nerve (SN), skeletal muscle (SM), brain (Br) and spinal cord (SC) after different infusion regimens. Data are shown as mean ± standard deviation, N=3-6 biological replicates. **p*<0.05, ***p*<0.01, indicates a significant difference in K_p,tissue_ between the tissues based on an ordinary one-way ANOVA test with Tukey’s multiple comparisons


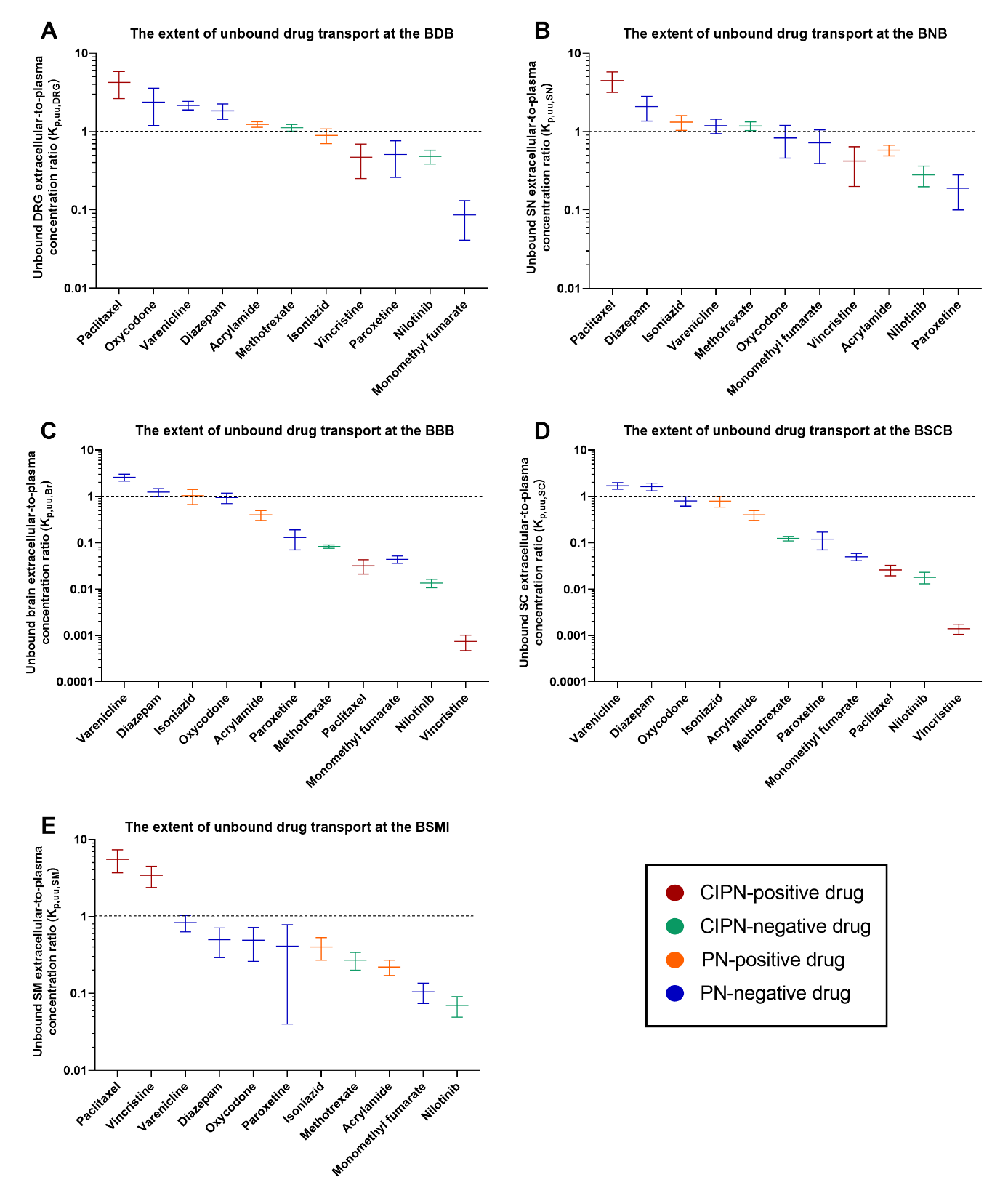


**Figure S3.**

Scatter plot of unbound tissue extracellular-to-plasma concentration ratio (K_p,uu_) of 11 selected drugs, sorted by mean K_p,uu_ from high to low, in dorsal root ganglia (DRG), sciatic nerve (SN), brain (Br), spinal cord (SC) or skeletal muscle (SM), describing the extent of unbound drug transport across the BDB **(A)**, BNB **(B)**, BBB **(C)**, BSCB **(D)** and BSMI **(E)**, respectively. K_p,uu_ = 1 is indicated as a black dashed line, describing predominant passive diffusion or mutually compensated influx and efflux transport. K_p,uu_ < 1 indicates predominant active efflux, while K_p,uu_ > 1 indicates predominant active uptake. Data presented as mean with standard deviation estimated using error propagation method ([51](#biblioRef050)). NB: note the differences in the scale.


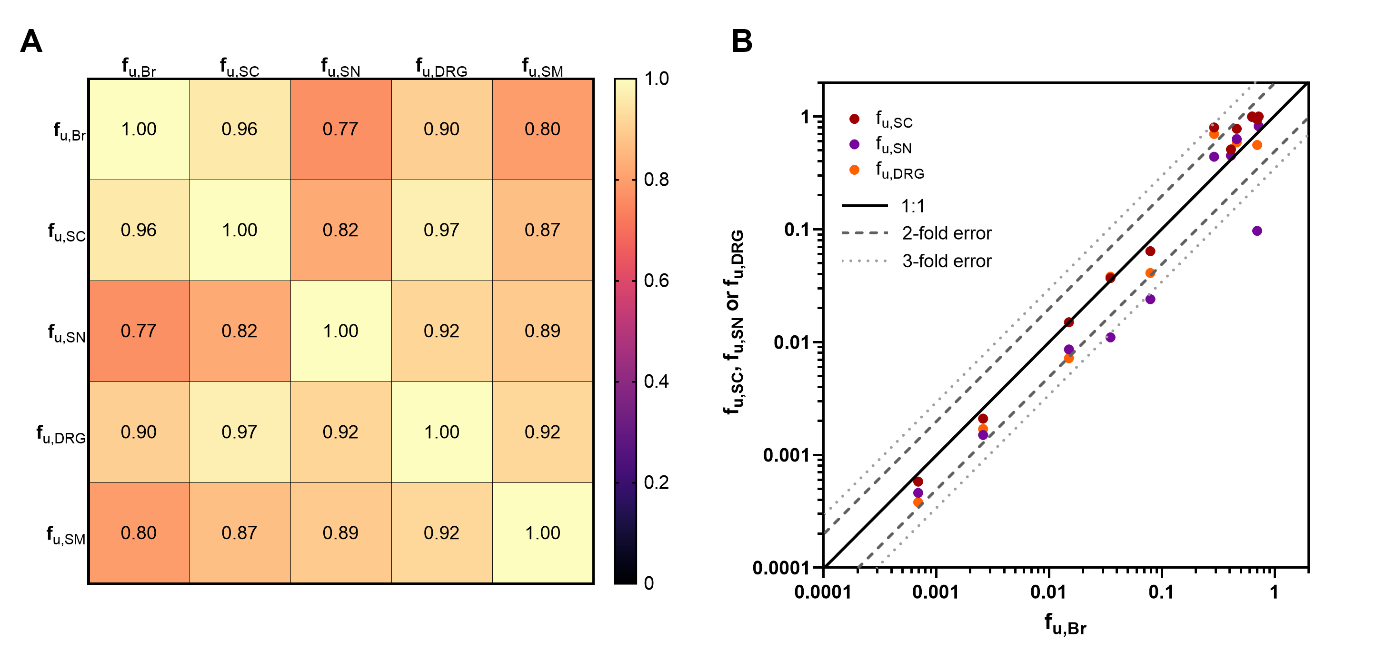


**Figure S4.**

**(A)** A heatmap of the correlation matrix for fraction of unbound drug in neural tissues and skeletal muscle. The correlation coefficient (r) was computed between unbound fraction in brain (f_u,Br_), spinal cord (f_u,SC_), sciatic nerve (f_u,SN_) and dorsal root ganglia (f_u,DRG_). **(B)** The relationship between unbound fraction in brain and unbound fraction in the other three neural tissues.


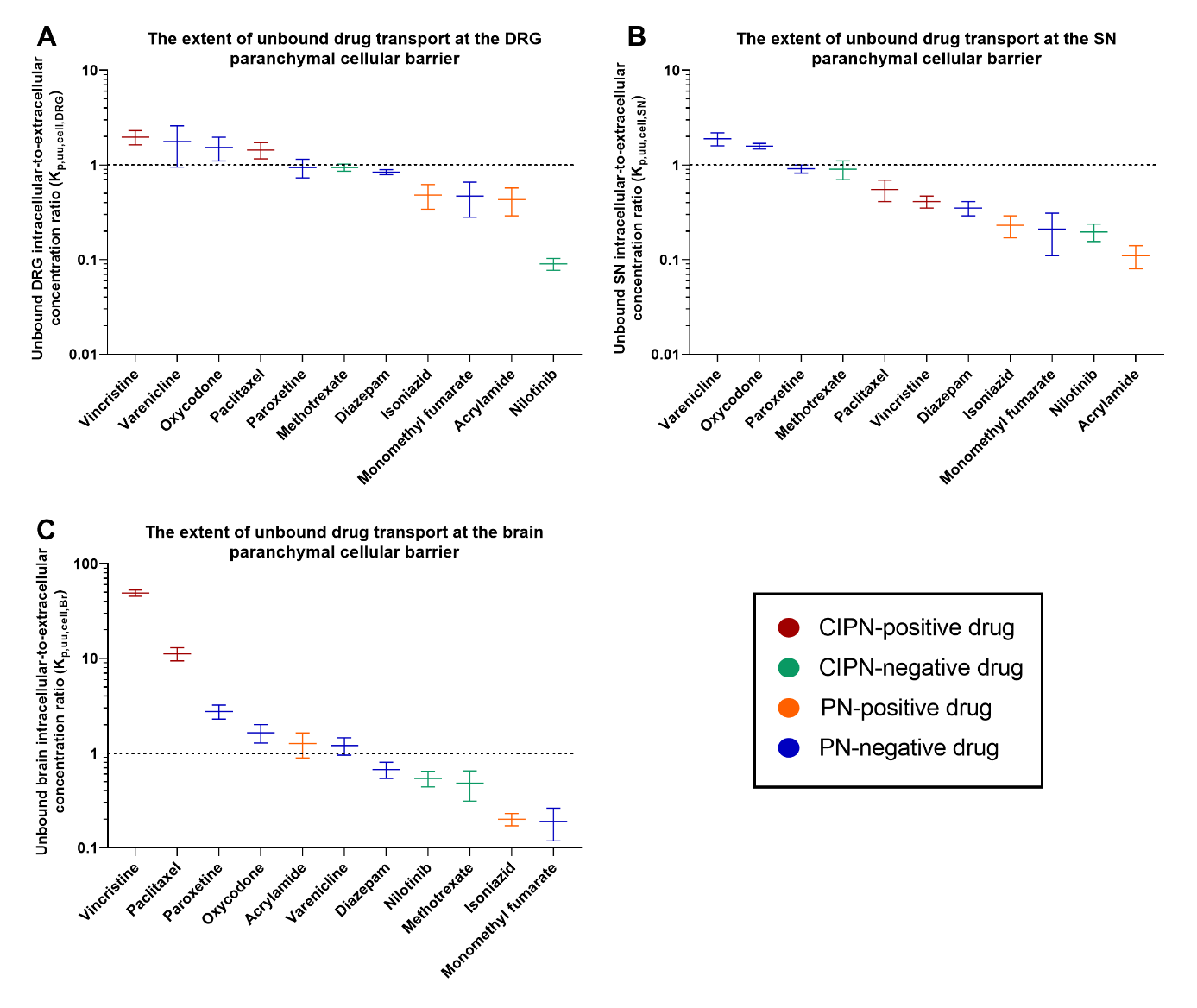


**Figure S5.**

Scatter plot of unbound tissue intracellular-to-extracellular concentration ratio (K_p,uu,cell_) of 11 selected drugs, sorted by mean K_p,uu_ from high to low, in dorsal root ganglia (DRG), sciatic nerve (SN) or brain (Br), describing the extent of unbound drug transport across the DRG **(A)**, SN **(B)**, or brain **(C)** parenchymal cellular barrier, respectively. K_p,uu,cell_ = 1 is indicated as a black dashed line, describing predominant passive diffusion or balance influx and efflux transport. K_p,uu,cell_ < 1 indicates limited intracellular entry, while K_p,uu,cell_ > 1 indicates active intracellular accumulation. Data presented as mean with standard deviation estimated using error propagation method. NB: note the differences in the scale.

**
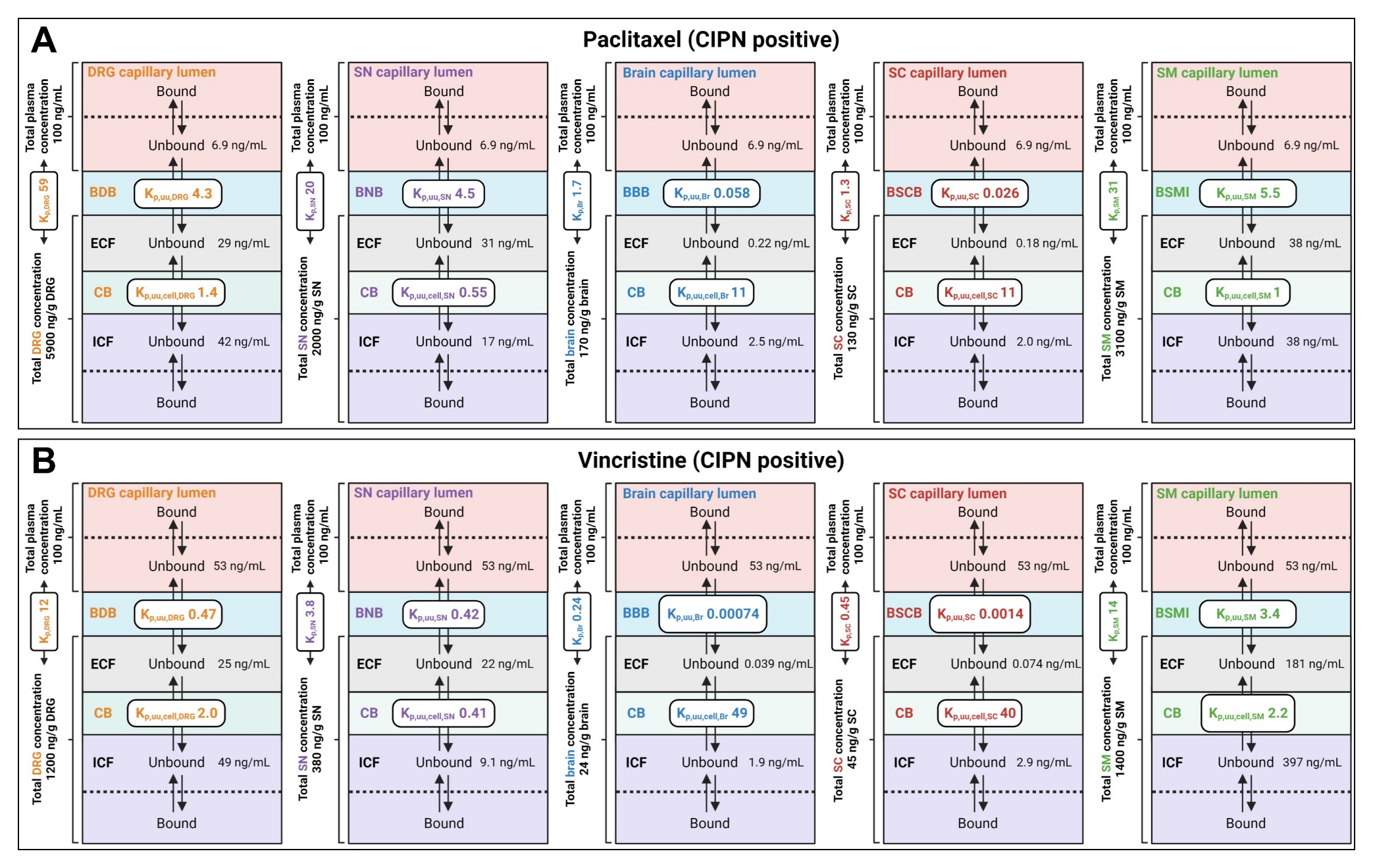
**

**Figure S6.**

Estimated steady-state unbound concentrations of paclitaxel (A) and vincristine (B) in the plasma, and in the extracellular (ECF) and intracellular (ICF) spaces of the dorsal root ganglia (DRG), sciatic nerve (SN), and brain (Br), spinal cord (SC) and skeletal muscle (SM) with total drug concentrations in the respective tissues indicated. f_u,plasma_, K_p_, K_p,uu_, and K_p,uu,cell_ measured in the current study were used for the calculations, with the assumption of the total plasma concentration of 100 ng/mL for each drug. See Supplementary Table S8 for the results of the simulation exercise for all drugs tested in the current study.
