# Supplementary material for: Analysis of the contributing role of drug transport across biological barriers in the development and treatment of chemotherapy-induced peripheral neuropathy
Source: Fluids Barriers CNS. 2024 Feb 8;21:13. doi: 10.1186/s12987-024-00519-7 (PMC10854123; doi:10.1186/s12987-024-00519-7)
Supplement: Supplementary file 2 — Additional file 2. Supplementary Tables S1-S7 | S1-S7 tables with titles. [file 12987_2024_519_MOESM2_ESM.docx]

**Supplementary Material: Supplementary Tables S1- S7**

**Analysis of the Contributing Role of Drug Transport Across Biological Barriers in the Development and Treatment of Chemotherapy-Induced Peripheral Neuropathy**

Yang Hu^1,2^, Milda Girdenyté^1,3^, Lieke Roest^1^, Iida Liukkonen^1^, Maria Siskou^1^, Frida Bällgren^1^, Margareta Hammarlund-Udenaes^1^, and Irena Loryan^1,^*

^1^ Translational Pharmacokinetics-Pharmacodynamics group, tPKPD, Department of Pharmacy, Faculty of Pharmacy, Uppsala University, Box 580, SE-751 23, Uppsala, Sweden

^2^ Current affiliation: Discovery ADME, Drug Discovery Sciences, Boehringer Ingelheim RCV GmbH & Co KG, A-1121, Vienna, Austria

^3^ Pharmacy and Pharmacology Center, Institute of Biomedical Sciences, Faculty of Medicine, Vilnius University, M.K. Čiurlionio, Str. 21/27, LT-03101 Vilnius, Lithuania

* Corresponding author:

Irena Loryan, MD, PhD

Translational PKPD group, Department of Pharmacy

Box 580, 751 23 Uppsala, Sweden

**Table S1**. Physicochemical properties including molecular weight, cLog P, pKa and ion class (information was extracted from DrugBank) and transporter liabilities of the selected drugs.

| Compound | Molecular weight (g/mol) | cLog P | pKa | | Ion class | Transporter substrate (rodent data) | |
| --- | --- | --- | --- | --- | --- | --- | --- |
|  |  |  | Strongest acid | Strongest base |  | Influx | Efflux |
| Paclitaxel | 853.9 | 3 | 11.9 | -1.2 | neutral | Oatp 1b2 ([1](#biblioRef00)), Oat 2 ([2](#biblioRef01)) | Mdr1 (P-gp) ([3](#biblioRef02)), Mrp2 ([4](#biblioRef03)) |
| Vincristine | 825 | 2.82 | 10.85 | 8.66 | neutral | N.I. | Mdr1 (P-gp) ([5](#biblioRef04)), Mrp1 ([6](#biblioRef05)), Mrp2 ([7](#biblioRef06)) |
| Methotrexate | 454.4 | -1.85 | 2.95 | 14.55 | acid | N.I. | Bcrp ([8](#biblioRef07)), Mrp3 ([9](#biblioRef08)), Mrp4 ([10](#biblioRef09)), Mrp5 ([11](#biblioRef010)), Oat1 ([12](#biblioRef011)), Oat3 ([13](#biblioRef012)) |
| Nilotinib | 529.5 | 4.9 | 12.38 | 5.92 | acid | N.I. | Mdr1 (P-gp) ([14](#biblioRef013)), Bcrp ([15](#biblioRef014)) |
| Isoniazid | 137.1 | -0.7 | 13.61 | 3.35 | neutral | N.I. | N.I. |
| Acrylamide | 71.1 | -0.67 | 16.7 | 0 | neutral | N.I. | N.I. |
| Varenicline | 211.3 | 0.9 |  | 9.73 | base | H^+^/OC antiporter ([16](#biblioRef015)), Oct 2 ([17](#biblioRef016)) | Mate ([18](#biblioRef017)) |
| Oxycodone | 315.4 | 0.7 |  | 9.1 | base | H^+^/OC antiporter ([19](#biblioRef018)) | N.I. |
| Paroxetine | 329.4 | 3.5 |  | 9.77 | base | N.I. | Mdr1 (P-gp) ([20](#biblioRef019)) |
| Monomethyl fumarate | 130.1 | 0.4 | 3.12 | -6.8 | acid | N.I. | N.I. |
| Diazepam | 284.7 | 2.82 |  | 2.92 | neutral | N.I. | N.I. |

N.I. Not identified

1. Leblanc AF, Sprowl JA, Alberti P, Chiorazzi A, Arnold WD, Gibson AA, et al. OATP1B2 deficiency protects against paclitaxel-induced neurotoxicity. J Clin Invest [Internet]. 2018;128:816–25. Available from: <https://www.ncbi.nlm.nih.gov/pubmed/29337310>

2. Marada VV, Florl S, Kuhne A, Muller J, Burckhardt G, Hagos Y. Interaction of human organic anion transporter 2 (OAT2) and sodium taurocholate cotransporting polypeptide (NTCP) with antineoplastic drugs. Pharmacol Res [Internet]. 2015;91:78–87. Available from: <https://www.ncbi.nlm.nih.gov/pubmed/25481222>

3. Kemper EM, Zandbergen AE van, Cleypool C, Mos HA, Boogerd W, Beijnen JH, et al. Increased penetration of paclitaxel into the brain by inhibition of P-Glycoprotein. Clin Cancer Res [Internet]. 2003;9:2849–55. Available from: <https://www.ncbi.nlm.nih.gov/pubmed/12855665>

4. Huisman MT, Chhatta AA, Tellingen O van, Beijnen JH, Schinkel AH. MRP2 (ABCC2) transports taxanes and confers paclitaxel resistance and both processes are stimulated by probenecid. Int J Cancer [Internet]. 2005;116:824–9. Available from: <https://www.ncbi.nlm.nih.gov/pubmed/15849751>

5. Liu J, Liu Y, Powell DA, Waalkes MP, Klaassen CD. Multidrug-resistance mdr1a/1b double knockout mice are more sensitive than wild type mice to acute arsenic toxicity, with higher arsenic accumulation in tissues. Toxicology [Internet]. 2002;170:55–62. Available from: <https://www.ncbi.nlm.nih.gov/pubmed/11750083>

6. Allen JD, Brinkhuis RF, Deemter L van, Wijnholds J, Schinkel AH. Extensive contribution of the multidrug transporters P-glycoprotein and Mrp1 to basal drug resistance. Cancer Res [Internet]. 2000;60:5761–6. Available from: <https://www.ncbi.nlm.nih.gov/pubmed/11059771>

7. Schinkel AH, Jonker JW. Mammalian drug efflux transporters of the ATP binding cassette (ABC) family: an overview. Adv Drug Deliver Rev [Internet]. 2003;55:3–29. Available from: <https://www.ncbi.nlm.nih.gov/pubmed/12535572>

8. Li L, Agarwal S, Elmquist WF. Brain efflux index to investigate the influence of active efflux on brain distribution of pemetrexed and methotrexate. Drug Metab Dispos [Internet]. 2013;41:659–67. Available from: <https://www.ncbi.nlm.nih.gov/pubmed/23297298>

9. Kitamura Y, Hirouchi M, Kusuhara H, Schuetz JD, Sugiyama Y. Increasing systemic exposure of methotrexate by active efflux mediated by multidrug resistance-associated protein 3 (mrp3/abcc3). J Pharmacol Exp Ther [Internet]. 2008;327:465–73. Available from: <https://www.ncbi.nlm.nih.gov/pubmed/18719291>

10. Sane R, Wu SP, Zhang R, Gallo JM. The effect of ABCG2 and ABCC4 on the pharmacokinetics of methotrexate in the brain. Drug Metab Dispos [Internet]. 2014;42:537–40. Available from: <https://www.ncbi.nlm.nih.gov/pubmed/24464805>

11. Jansen RS, Mahakena S, Haas M de, Borst P, Wetering K van de. ATP-binding Cassette Subfamily C Member 5 (ABCC5) Functions as an Efflux Transporter of Glutamate Conjugates and Analogs. J Biol Chem [Internet]. 2015;290:30429–40. Available from: <https://www.ncbi.nlm.nih.gov/pubmed/26515061>

12. Uwai Y, Saito H, Inui K. Interaction between methotrexate and nonsteroidal anti-inflammatory drugs in organic anion transporter. Eur J Pharmacol [Internet]. 2000;409:31–6. Available from: <https://www.ncbi.nlm.nih.gov/pubmed/11099697>

13. VanWert AL, Sweet DH. Impaired clearance of methotrexate in organic anion transporter 3 (Slc22a8) knockout mice: a gender specific impact of reduced folates. Pharmaceut Res [Internet]. 2008;25:453–62. Available from: <https://www.ncbi.nlm.nih.gov/pubmed/17660957>

14. Shukla S, Robey RW, Bates SE, Ambudkar SV. Sunitinib (Sutent, SU11248), a small-molecule receptor tyrosine kinase inhibitor, blocks function of the ATP-binding cassette (ABC) transporters P-glycoprotein (ABCB1) and ABCG2. Drug Metab Dispos [Internet]. 2009;37:359–65. Available from: <https://www.ncbi.nlm.nih.gov/pubmed/18971320>

15. Hegedus C, Ozvegy-Laczka C, Apati A, Magocsi M, Nemet K, Orfi L, et al. Interaction of nilotinib, dasatinib and bosutinib with ABCB1 and ABCG2: implications for altered anti-cancer effects and pharmacological properties. Brit J Pharmacol [Internet]. 2009;158:1153–64. Available from: <https://www.ncbi.nlm.nih.gov/pubmed/19785662>

16. Kurosawa T, Higuchi K, Okura T, Kobayashi K, Kusuhara H, Deguchi Y. Involvement of Proton-Coupled Organic Cation Antiporter in Varenicline Transport at Blood-Brain Barrier of Rats and in Human Brain Capillary Endothelial Cells. J Pharm Sci [Internet]. 2017;106:2576–82. Available from: <https://www.ncbi.nlm.nih.gov/pubmed/28454746>

17. Feng B, Obach RS, Burstein AH, Clark DJ, Morais SM de, Faessel HM. Effect of human renal cationic transporter inhibition on the pharmacokinetics of varenicline, a new therapy for smoking cessation: an in vitro-in vivo study. Clin Pharmacol Ther [Internet]. 2008;83:567–76. Available from: <https://www.ncbi.nlm.nih.gov/pubmed/17971819>

18. Kajiwara M, Masuda S, Watanabe S, Terada T, Katsura T, Inui K. Renal tubular secretion of varenicline by multidrug and toxin extrusion (MATE) transporters. Drug Metab Pharmacok [Internet]. 2012;27:563–9. Available from: <https://www.ncbi.nlm.nih.gov/pubmed/22510868>

19. Shimomura K, Okura T, Kato S, Couraud PO, Schermann JM, Terasaki T, et al. Functional expression of a proton-coupled organic cation (H+/OC) antiporter in human brain capillary endothelial cell line hCMEC/D3, a human blood-brain barrier model. Fluids Barriers Cns [Internet]. 2013;10:8. Available from: <https://www.ncbi.nlm.nih.gov/pubmed/23351963>

20. Kowalska M, Nowaczyk J, Fijalkowski L, Nowaczyk A. Paroxetine-Overview of the Molecular Mechanisms of Action. Int J Mol Sci [Internet]. 2021;22:1662. Available from: <https://www.ncbi.nlm.nih.gov/pubmed/33562229>

**Table S2.** The dosing regimens (period, intravenous infusion rate), drug concentration in the infusion solution and vehicles for the selected drugs in the pharmacokinetic studies in rats.

| Compound | Animal sex and species | Number of animals (N) | Vehicle | Drug concentration in the infusion solution (mg/mL) | Relevant human therapeutic concentration (ng/mL) | Targeted steady-state total plasma concentration (ng/mL) ^a^ | Systemic clearance used for simulation (L/h/kg) | Dosing regimen | | | |
| --- | --- | --- | --- | --- | --- | --- | --- | --- | --- | --- | --- |
|  |  |  |  |  |  |  |  | Loading infusion | | Maintenance infusion | |
|  |  |  |  |  |  |  |  | Period  (h) | Rate (mg/h/kg) | Period  (h) | Rate (mg/h/kg) |
| Paclitaxel | Male Wistar-Han rats | N=4 | Cremophor:Ethanol:Saline (5:5:90, v/v/v) | 0.6 | 146 (1,2) | 184 | 1.9 ^b^ | 0-0.5 | 6.3 | 0.5-4 | 0.37 |
|  | Male Wistar-Han rats | N=3 | Cremophor:Ethanol:Saline (0.4:0.4:99.2, v/v/v) | 0.05 |  | 15 |  | 0-0.25 | 0.33 | 0.25-4 | 0.027 |
|  | Male Wistar-Han rats | N=5 | Cremophor:Ethanol:Saline (1:1:2, v/v/v) | 3 |  | N.A. |  | N.A. | N.A. | 0-240 | 0.05 |
| Vincristine | Male Wistar-Han rats | N=3 | Saline with 1.5 mg/mL mannitol | 0.015 | 3.1 (3) | 4.6 | 1.7 ^b^ | 0-0.25 | 0.17 | 0.25-4 | 0.012 |
|  | Male Wistar-Han rats | N=3 | Water for injection with 100 mg/mL mannitol | 1 |  | N.A. |  | N.A. | N.A. | 0-48 | 0.017 |
| Methotrexate | Male Wistar-Han rats | N=6 | Saline | 2 | 50-1000 (4) | 1000 | 1.3 (5) | 0-0.5 | 2.4 | 0.5-4 | 1.4 |
| Nilotinib | Male Sprague-Dawley rats | N=6 | DMSO:Milli-Q water  (3:97, v/v) | 1.5 | 830-1700 (6,7) | 2000 | 0.39 ^b^ | 0-0.25 | 20 | 0.25-4 | 0.78 |
| Isoniazid | Male Sprague-Dawley rats | N=6 | Saline | 3.2 | 3000-6000 (8,9) | 4000 | 0.96 ^b^ | N.A. | N.A. | 0-4 | 3.8 |
| Acrylamide | Male Sprague-Dawley rats | N=6 | Saline | 0.3 | N.A. | 1000 | 0.31 (10) | 0-0.25 | 2.5 | 0.25-4 | 0.31 |
| Varenicline | Male Sprague-Dawley rats | N=5 | Saline | 0.05 | 10 (11) | 8 | 1.5 ^b^ | 0-0.25 | 0.40 | 0.25-4 | 0.02 |
| Oxycodone | Male Sprague-Dawley rats | N=5 | Saline | 0.2 | 12-87 (12) | 40 | 7.5 (13) | N.A. | N.A. | 0-4 | 0.3 |
| Paroxetine | Male Sprague-Dawley rats | N=6 | Milli-Q water | 0.2 | 27.5 (14) | 27.5 | 4.5 ^b^ | 0-0.25 | 1.8 | 0.25-4 | 0.12 |
| Monomethyl fumarate | Male Sprague-Dawley rats | N=6 | Milli-Q water | 5 | N.A. | 5000 | 6.9 ^b^ | N.A. | N.A. | 0-4 h | 8.3 |
| Diazepam | Male Sprague-Dawley rats | N=6 | Ethanol:Saline  (2.5:97.5, v/v) | 0.25 | 300 (15) | 50 | 3.7 (16) | 0-0.25 | 0.83 | 0.25-4 | 0.18 |

N.A. Not available

^a^ considered the differences between plasma protein binding between humans and rats

^b^ Obtained from a IV 10 min infusion PK study performed in-house

1. Stage TB, Bergmann TK, Kroetz DL. Clinical Pharmacokinetics of Paclitaxel Monotherapy: An Updated Literature Review. Clin Pharmacokinet **2018**;57:7-19

2. Sparreboom A, Scripture CD, Trieu V, Williams PJ, De T, Yang A*, et al.* Comparative preclinical and clinical pharmacokinetics of a cremophor-free, nanoparticle albumin-bound paclitaxel (ABI-007) and paclitaxel formulated in Cremophor (Taxol). Clin Cancer Res **2005**;11:4136-43

3. Yang F, Jiang M, Lu M, Hu P, Wang H, Jiang J. Pharmacokinetic Behavior of Vincristine and Safety Following Intravenous Administration of Vincristine Sulfate Liposome Injection in Chinese Patients With Malignant Lymphoma. Front Pharmacol **2018**;9:991

4. Methotrexate monograph <https://pdf.hres.ca/dpd_pm/00023475.PDF>. **2014**

5. Hu Y, Rip J, Gaillard PJ, de Lange ECM, Hammarlund-Udenaes M. The Impact of Liposomal Formulations on the Release and Brain Delivery of Methotrexate: An In Vivo Microdialysis Study. J Pharm Sci **2017**;106:2606-13

6. Tanaka C, Yin OQ, Sethuraman V, Smith T, Wang X, Grouss K*, et al.* Clinical pharmacokinetics of the BCR-ABL tyrosine kinase inhibitor nilotinib. Clin Pharmacol Ther **2010**;87:197-203

7. Kim KP, Ryu MH, Yoo C, Ryoo BY, Choi DR, Chang HM*, et al.* Nilotinib in patients with GIST who failed imatinib and sunitinib: importance of prior surgery on drug bioavailability. Cancer Chemother Pharmacol **2011**;68:285-91

8. Alsultan A, Peloquin CA. Therapeutic drug monitoring in the treatment of tuberculosis: an update. Drugs **2014**;74:839-54

9. Prahl JB, Johansen IS, Cohen AS, Frimodt-Moller N, Andersen AB. Clinical significance of 2 h plasma concentrations of first-line anti-tuberculosis drugs: a prospective observational study. J Antimicrob Chemother **2014**;69:2841-7

10. Kim TH, Shin S, Kim KB, Seo WS, Shin JC, Choi JH*, et al.* Determination of acrylamide and glycidamide in various biological matrices by liquid chromatography-tandem mass spectrometry and its application to a pharmacokinetic study. Talanta **2015**;131:46-54

11. Faessel HM, Gibbs MA, Clark DJ, Rohrbacher K, Stolar M, Burstein AH. Multiple-dose pharmacokinetics of the selective nicotinic receptor partial agonist, varenicline, in healthy smokers. J Clin Pharmacol **2006**;46:1439-48

12. Oxycodone monograph <https://pdf.hres.ca/dpd_pm/00046337.PDF>. **2018**

13. Bostrom E, Simonsson US, Hammarlund-Udenaes M. In vivo blood-brain barrier transport of oxycodone in the rat: indications for active influx and implications for pharmacokinetics/pharmacodynamics. Drug Metab Dispos **2006**;34:1624-31

14. Paroxetine monograph <https://pdf.hres.ca/dpd_pm/00039978.PDF>. **2017**

15. Eatman FB, Colburn WA, Boxenbaum HG, Posmanter HN, Weinfeld RE, Ronfeld R*, et al.* Pharmacokinetics of diazepam following multiple-dose oral administration to healthy human subjects. J Pharmacokinet Biopharm **1977**;5:481-94

16. Dhir A, Rogawski MA. Determination of minimal steady-state plasma level of diazepam causing seizure threshold elevation in rats. Epilepsia **2018**;59:935-44

**Table S3.** Summary of the dosing regimens, total doses, steady-state total plasma concentrations (C_tot,ss,plasma_), unbound fraction in plasma (f_u,plasma_), tissue sampling time, and total tissue-to-plasma concentration ratios at steady-state (K_p,tissue,ss_) of the selected drugs (N, biological replicates; n, techinique replicates).

| Compound | Dosing regimen | Total dose | C_tot,ss,plasma_ (ng/mL) | f_u,plasma_ | Tissue sampling time | Total tissue-to-plasma concentration ratio at steady-state (K_p,tissue,ss_) | | | | |
| --- | --- | --- | --- | --- | --- | --- | --- | --- | --- | --- |
|  |  |  |  |  |  | Dorsal root ganglia (DRG) | Sciatic nerve (SN) | Brain  (Br) | Spinal cord (SC) | Skeletal muscle (SM) |
| Paclitaxel | IV 4h constant infusion  (High dose) | 4.5 mg/kg | 220 ± 39  (N=4) | 0.069 ± 0.0062 (N=6, n=1 or 2) | 4h | 2.8 ± 1.5  (N=4) | 3.6 ± 0.92  (N=4) | 0.21 ± 0.072  (N=4) | 0.12 ± 0.021  (N=4) | 7.8 ± 5.2  (N=4) |
|  | IV 4h constant infusion  (Low dose) | 0.18 mg/kg | 5.3 ± 0.26 (N=3) |  | 4h | 5.5 ± 0.85  (N=3) | 3.9 ± 1.2  (N=3) | 0.33 ± 0.28  (N=3) | N.D. | 8.8 ± 1.5  (N=3) |
|  | SC 240h constant infusion via ALZET pump | 12 mg/kg | 12 ± 4.1  (N=5) |  | 240h | 59 ± 26  (N=5) | 20 ± 6.4  (N=3) | 1.7 ± 0.62  (N=5) | 1.3 ± 0.38  (N=5) | 31 ± 12  (N=5) |
| Vincristine | IV 4h constant infusion | 0.087 mg/kg | 3.9 ± 0.59  (N=3) | 0.53 ± 0.029 (N=3, n=2) | 4h | 5.3 ± 1.1  (N=3) | 2.3 ± 0.24  (N=3) | 0.082 ± 0.015  (N=3) | 0.17 ± 0.11  (N=3) | 6.8 ± 1.2  (N=3) |
|  | SC 48 constant infusion via ALZET pump | 0.82 mg/kg | 10 ± 0.35  (N=2) |  | 48h | 12 ± 6.2  (N=3) | 3.8 ± 2.1  (N=3) | 0.24 ± 0.10  (N=3) | 0.45 ± 0.13  (N=3) | 14 ± 4.8  (N=3) |
| Methotrexate | IV 4h constant infusion | 6.1 mg/kg | 880 ± 160  (N=6) | 0.39 ± 0.018 (N=6, n=1 or 2) | 4h | 0.41 ± 0.045  (N=6) | 0.46 ± 0.067  (N=6) | 0.024 ± 0.0023  (N=6) | 0.036 ± 0.0048  (N=6) | 0.13 ± 0.038  (N=6) |
| Nilotinib | IV 4h constant infusion | 7.8 mg/kg | 830 ± 110  (N=6) | 0.0065 ± 0.0017 (N=3, n=1 or 2) | 4h | 0.73 ± 0.089  (N=6) | 0.79 ± 0.25  (N=6) | 0.069 ± 0.010  (N=6) | 0.091 ± 0.028  (N=6) | 0.49 ± 0.17  (N=6) |
| Isoniazid | IV 4h constant infusion | 15 mg/kg | 710 ± 160 (N=6) | 0.93 ± 0.074 (N=3, n=2) | 4h | 0.57 ± 0.14  (N=5) | 0.64 ± 0.16 (N=6) | 0.67 ± 0.26  (N=5) | 0.51 ± 0.14 (N=6) | 0.58 ± 0.15 (N=5) |
| Acrylamide | IV 4h constant infusion | 1.8 mg/kg | 830 ± 61 (N=6) | 0.96 ± 0.033 (N=3, n=2) | 4h | 0.90 ± 0.072  (N=5) | 0.61 ± 0.094 (N=6) | 0.69 ± 0.075 (N=6) | 0.69 ± 0.084 (N=6) | 0.69 ± 0.14 (N=6) |
| Varenicline | IV 4h constant infusion | 0.075 mg/kg | 15 ± 1.3 (N=5) | 0.92 ± 0.035 (N=6, n=1 or 2) | 4h | 6.0 ± 0.85  (N=5) | 3.3 ± 0.74 (N=5) | 6.2 ± 1.2  (N=5) | 4.1 ± 0.70  (N=5) | 4.1 ± 1.1  (N=5) |
| Oxycodone | IV 4h constant infusion | 1.2 mg/kg | 80 ± 41 (N=5) | 0.89 ± 0.026 (N=6, n=1 or 2) | 4h | 7.2 ± 3.8  (N=5) | 2.6 ± 1.2 (N=5) | 3.3 ± 0.92  (N=5) | 2.9 ± 0.70  (N=5) | 2.6 ± 1.3  (N=5) |
| Paroxetine | IV 4h constant infusion | 0.91 mg/kg | 29 ± 7.9 (N=6) | 0.086 ± 0.0070 (N=6, n=1 or 2) | 4h | 24 ± 14  (N=6) | 11 ± 5.6 (N=6) | 12 ± 5.6  (N=6) | 11 ± 5.2  (N=6) | 14 ± 13  (N=6) |
| Monomethyl fumarate | IV 4h constant infusion | 33 mg/kg | 2500 ± 730 (N=6) | 0.80 ± 0.058 (N=6, n=1 or 2) | 4h | 0.039 ± 0.023  (N=6) | 0.14 ± 0.032 (N=6) | 0.0092 ± 0.0011  (N=6) | 0.010 ± 0.0017  (N=6) | 0.055 ± 0.018  (N=6) |
| Diazepam | IV 4h constant infusion | 0.75 mg/kg | 34 ± 12 (N=6) | 0.16 ± 0.038 (N=3, n=2) | 4h | 6.5 ± 1.5  (N=6) | 11 ± 4.3 (N=6) | 3.8 ± 0.40  (N=6) | 5.0 ± 0.65  (N=6) | 1.4 ± 0.68  (N=6) |

**Table S4.** Unbound fraction (f_u_) and unbound volume of distribution (V_u_) for the selected drugs in dorsal root ganglia, sciatic nerve, brain, spinal cord and skeletal muscle. Data are expressed as mean ± standard deviation (N, biological replicates, n, technical replicates).

| Compound | Unbound fraction (f_u_) | | | | |  | | Unbound volume of distribution (V_u_, mL/g tissue) | | | |
| --- | --- | --- | --- | --- | --- | --- | --- | --- | --- | --- | --- |
|  | Dorsal root ganglia | Sciatic nerve | Brain | Spinal cord | Skeletal muscle | |  | | Dorsal root ganglia | Sciatic nerve | Brain |
| Paclitaxel | 0.0072 ± 0.00095 (N=3, n=1) | 0.0086 ± 0.00078  (N=3, n=1) | 0.015 ± 0.0028  (N=5, n=1) | 0.015 ± 0.0016  (N=5, n=1 or 2) | 0.013 ± 0.0027  (N=6, n=1) | |  | | 200 ± 44  (N=8, n=1) | 64 ± 18  (n=8, n=2) | 745 ± 75  (N=3, n=5) |
| Vincristine | 0.041 ± 0.0080  (N=3, n=1) | 0.024 ± 0.0041  (N=3, n=1) | 0.079 ± 0.0063  (N=3, n=2) | 0.064 ± 0.0056  (N=3, n=2) | 0.3 ± 0.099  (N=3, n=2) | |  | | 48 ± 7.0  (N=4, n=1) | 17 ± 2.0  (N=4, n=1 or 2) | 620 ± 45  (N=3, n=5) |
| Methotrexate | 1.0 ± 0.0  (N=3, n=1) | 0.99 ± 0.015  (N=3, n=1 or 2) | 0.63 ± 0.24  (N=9, n=1 or 2) | 1.0 ± 0.0  (N=3, n=1 or 2) | 0.84 ± 0.18  (N=3, n=2) | |  | | 0.94 ± 0.08  (N=3, n=2) | 1.0 ± 0.06  (N=3, n=2) | 0.75 ± 0.053  (N=3, n=5) |
| Nilotinib | 0.00041, 0.00036  (N=2, n=1) | 0.00046 ± 0.000099  (N=6, n=1 or 2) | 0.00069 ± 0.000092  (N=3, n=2) | 0.00058 ± 0.000061  (N=4, n=2) | 0.00098 ± 0.00013  (N=2, n=2) | |  | | 237 ± 39  (N=6, n=1) | 427 ± 88  (N=6, n=1) | 788 ± 157  (N=3, n=5) |
| Isoniazid | 0.70 ± 0.23  (N=3, n=1) | 0.44 ± 0.14  (N=3, n=2) | 0.29 ± 0.051  (N=3, n=2) | 0.80 ± 0.17  (N=3, n=2) | 0.74 ± 0.29  (N=3, n=2) | |  | | 0.69 ± 0.06  (N=3, n=2) | 0.52 ± 0.09  (N=3, n=2) | 0.69 ± 0.07  (N=3, n=4 or 5) |
| Acrylamide | 0.56 ± 0.21  (N=3, n=1) | 0.097 ± 0.032  (N=2, n=2) | 0.70 ± 0.20  (N=3, n=2) | 0.94 ± 0.11  (N=3, n=2) | 0.33 ± 0.12  (N=2, n=2) | |  | | 0.76 ± 0.07  (N=3, n=2) | 1.1 ± 0.20  (N=3, n=2) | 1.8 ± 0.53  (N=3, n=5) |
| Varenicline | 0.59 ± 0.29  (N=3, n=1) | 0.63 ± 0.11  (N=3, n=1 or 2) | 0.46 ± 0.11  (N=6, n=1 or 2) | 0.78 ± 0.17  (N=3, n=2) | 0.42 ± 0.054  (N=3, n=2) | |  | | 3.0 ± 0.21  (N=3, n=2) | 3.0 ± 0.12  (N=3, n=2) | 2.6 ± 0.42  (N=3, n=4 or 5) |
| Oxycodone | 0.45 ± 0.14  (N=3, n=1) | 0.45 ± 0.035  (N=3, n=1 or 2) | 0.41 ± 0.10  (N=6, n=1 or 2) | 0.51 ± 0.045  (N=3, n=2) | 0.40 ± 0.047  (N=3, n=2) | |  | | 3.4 ± 0.28  (N=3, n=2) | 3.5 ± 0.12  (N=3, n=2) | 4.0 ± 0.26  (N=3, n=4 or 5) |
| Paroxetine | 0.0017 ± 0.00031 (N=3, n=1) | 0.0015 ± 0.000055  (N=3, n=1 or 2) | 0.0026 ± 0.00050  (N=6, n=1 or 2) | 0.0021 ± 0.00023  (N=3, n=2) | 0.0060 ± 0.0012 (N=2, n=2) | |  | | 555 ± 139  (N=6, n=1) | 649 ± 69  (N=6, n=1) | 1058 ± 72  (N=3, n=4 or 5) |
| Monomethyl fumarate | 0.82 ± 0.28  (N=6, n=1) | 0.82 ± 0.29  (N=3, n=2) | 0.72 ± 0.32  (N=9, n=1 or 2) | 1.0 ± 0.0  (N=3, n=1 or 2) | 1.0 ± 0.0  (N=3, n=1 or 2) | |  | | 0.57 ± 0.26  (N=3, n=2) | 0.25 ± 0.14  (N=3, n=2) | 0.26 ± 0.06  (N=3, n=4 or 5) |
| Diazepam | 0.038 ± 0.0027  (N=3, n=1) | 0.011 ± 0.0022  (N=2, n=2) | 0.035 ± 0.0075  (N=3, n=1) | 0.037 ± 0.0036  (N=3, n=2) | 0.060 ± 0.012  (N=3, n=2) | |  | | 22 ± 1.2  (N=6, n=1) | 32 ± 4.0  (N=6, n=1 or 2) | 19 ± 1.0  (N=3, n=4 or 5) |

**Table S5.** Detailed sample preparation procedures for the selected drugs prior to UPLC-MS/MS analysis.

| Compound | Sample volume (μL) | Protein precipitation | Dilution |
| --- | --- | --- | --- |
| Paclitaxel | 50 | 150 μL acetonitrile with 50 ng/mL paclitaxel-D5 | 100 μL supernatant mixed with 100 μL 0.1% formic acid in water |
| Vincristine | 50 | 150 μL acetonitrile with 50 ng/mL vincristine-D3 | 100 μL supernatant mixed with 100 μL 0.1% formic acid in water |
| Methotrexate | 50 | 150 μL acetonitrile with 50 ng/mL methotrexate-D3 and 1% formic acid | 50 μL supernatant mixed with 200 μL 0.01% acetic acid in water |
| Nilotinib | 50 | 150 μL acetonitrile with 5 ng/mL nilotinib-D3 | 100 μL supernatant mixed with 100 μL 0.1% formic acid in water |
| Isoniazid | 50 | 150 μL acetonitrile with 500 ng/mL isoniazid-D4 | 5 μL supernatant mixed with 500 μL water |
| Acrylamide | 50 | 150 μL acetonitrile with 50 ng/mL acrylamide-D3 | 100 μL supernatant evaporated and reconstituted with 120 μL 0.1% formic acid in water |
| Varenicline | 50 | 150 μL acetonitrile with 100 ng/mL varenicline-D4 | 50 μL supernatant mixed with 200 μL 10 mM ammonium formate |
| Oxycodone | 50 | 150 μL acetonitrile with 10 ng/mL oxycodone-D6 | 50 μL supernatant mixed with 150 μL 0.1% formic acid |
| Paroxetine | 50 | 150 μL acetonitrile with 50 ng/mL paroxetine-D6 | 100 μL supernatant mixed with 200 μL 0.1% formic acid in water |
| Monomethyl fumarate | 50 | 150 μL acetonitrile with 25 ng/mL monomethyl fumarate-D3 and 1% formic acid | 50 μL supernatant mixed with 200 μL 0.01% acetic acid in water |
| Diazepam | 50 | 150 μL acetonitrile with 10 ng/mL diazepam-D5 | 50 μL supernatant mixed with 150 μL 0.1% formic acid in water |

**Table S6.** Detailed mass spectrometric conditions for the selected drugs.

| Analyte | Internal standard (IS) | ESI mode | Capillary voltage (kV) | Source temperature (°C) | Dissolvation temperature (°C) | Cone gas (L/hr) | Dissolvation gas  (L/hr) | Cone voltage (V) | Collision energy (V) | MRM transition | |
| --- | --- | --- | --- | --- | --- | --- | --- | --- | --- | --- | --- |
|  |  |  |  |  |  |  |  |  |  | Analyte | IS |
| Paclitaxel | Paclitaxel-D5 | Positive | 2 | 150 | 600 | 75 | 250 | 40 | 20 | 854.4→286.1 | 859.4→291.1 |
| Vincristine | Vincristine-D3 | Positive | 3 | 150 | 600 | 0 | 1000 | 50 | 16 | 413.4→362.2 | 414.9→362.8 |
| Methotrexate | Methotrexate-D3 | Positive | 3.5 | 150 | 600 | 30 | 1000 | 30 | 18 | 455.3→308.2 | 458.3→311.2 |
| Nilotinib | Nilotinib-D3 | Positive | 3 | 150 | 600 | 0 | 1000 | 30 | 30 | 530.3→289.2 | 533.3→289.2 |
| Isoniazid | Isoniazid-D4 | Positive | 1 | 150 | 600 | 30 | 1000 | 20 | 12 | 138.0→120.9 | 142.0→124.9 |
| Acrylamide | Acrylamide-D3 | Positive | 0.5 | 150 | 600 | 50 | 1000 | 20 | 10 | 72.0→54.9 | 75.0→57.9 |
| Varenicline | Varenicline-D4 | Positive | 3 | 150 | 600 | 0 | 1000 | 70 | 22 | 212.0→43.9 | 216.0→47.9 |
| Oxycodone | Oxycodone-D6 | Positive | 0.5 | 150 | 600 | 10 | 1000 | 10 | 19 | 316.1→298.1 | 322.2→304.1 |
| Paroxetine | Paroxetine-D6 | Positive | 3 | 150 | 600 | 0 | 1000 | 40 | 20 | 330.1→192.1 | 336.1→198.1 |
| Monomethyl fumarate | Monomethyl fumarate-D3 | Negative | 2 | 150 | 600 | 0 | 1000 | -10 | -18 | 128.9→30.8 | 131.9→33.8 |
| Diazepam | Diazepam-D5 | Positive | 3 | 150 | 600 | 30 | 1000 | 40 | 26 | 285.1→154.0 | 290.1→154.0 |

**Table S7.** Detailed chromatographic conditions for the selected drugs

| Analyte | Internal standard (IS) | Column | Column temperature (°C) | Mobile phase | | UPLC gradient | Run time (min) | Flow rate (mL/min) | Injection volume (μL) | Retention time (min) | |
| --- | --- | --- | --- | --- | --- | --- | --- | --- | --- | --- | --- |
|  |  |  |  | A (aqueous) | B  (organic) |  |  |  |  | Analyte | IS |
| Paclitaxel | Paclitaxel-D5 | Waters ACQUITY UPLC BEH C18 (2.1 × 50 mm, 1.7 μm) | 25 | 0.1% formic acid in water | 0.1% formic acid in acetonitrile | 0-2.5 min, 5-95% B; 2.5-3 min, 95% B; 3-3.2 min, 95-5% B | 3.5 | 0.3 | 10 | 2.38 | 2.37 |
| Vincristine | Vincristine-D3 | Waters ACQUITY UPLC BEH C18 (2.1 × 50 mm, 1.7 μm) | 25 | 0.1% formic acid in water | 0.1% formic acid in acetonitrile | 0-2.5 min, 5-95% B; 2.5-3 min, 95% B; 3-3.2 min, 95-5% B | 3.5 | 0.3 | 10 | 1.76 | 1.76 |
| Methotrexate | Methotrexate-D3 | Waters ACQUITY UPLC BEH C18 (2.1 × 50 mm, 1.7 μm) | 25 | 0.01% formic acid in water | 0.01% formic acid in acetonitrile | 0-1 min, 5% B; 1-2 min, 5-95% B; 2-2.5 min, 95% B; 2.5-2.7 min, 95-5% B | 3 | 0.3 | 5 | 1.99 | 1.98 |
| Nilotinib | Nilotinib-D3 | Waters ACQUITY UPLC BEH C18 (2.1 × 50 mm, 1.7 μm) | 25 | 0.1% formic acid in water | 0.1% formic acid in acetonitrile | 0-2.5 min, 5-95% B; 2.5-3 min, 95% B; 3-3.2 min, 95-5% B | 3.5 | 0.3 | 5 | 1.92 | 1.92 |
| Isoniazid | Isoniazid-D4 | Waters ACQUITY UPLC BEH C18 (2.1 × 50 mm, 1.7 μm) | 25 | 0.1% ammonia in water | 0.1% ammonia in acetonitrile | 0-1.5 min, 0.5% B; 1.5-2 min, 0.5-90% B; 2-3 min, 90% B; 3-3.2 min, 90-0.5% B | 4.5 | 0.3 | 5 | 1.32 | 1.3 |
| Acrylamide | Acrylamide-D3 | Waters ACQUITY UPLC HSS C18 (2.1 × 100 mm, 1.8 μm) | 40 | 0.1% formic acid in water | Methanol | 0-4 min, 1-5% B; 4-4.5 min, 5-95% B; 4.5-5.5 min, 95% B; 5.5-5.7 min, 95-1% B | 6.5 | 0.2 | 5 | 2.78 | 2.76 |
| Varenicline | Varenicline-D4 | Waters ACQUITY UPLC BEH C18 (2.1 × 50 mm, 1.7 μm) | 25 | 10 mM ammonium formate | 10% 10 mM ammonium formate and 90% acetonitrile | 0-1 min, 5% B; 1-1.5 min, 5-90% B; 1.5-2.5 min, 90% B; 2.5-2.7 min, 90-5% B | 3 | 0.3 | 5 | 1.94 | 1.94 |
| Oxycodone | Oxycodone-D6 | Waters ACQUITY UPLC BEH C18 (2.1 × 50 mm, 1.7 μm) | 40 | 0.1% formic acid in water | 0.1% formic acid in acetonitrile | 0-1 min, 5% B; 1-2.5 min, 5-45% B; 2.5-3.5 min, 45% B; 3.5-4 min, 45-90% B; 4.5-4.8 min, 90-5% B | 5.5 | 0.3 | 5 | 2.29 | 2.29 |
| Paroxetine | Paroxetine-D6 | Waters ACQUITY UPLC BEH C18 (2.1 × 50 mm, 1.7 μm) | 25 | 0.1% formic acid in water | 0.1% formic acid in acetonitrile | 0-2.5 min, 5-95% B; 2.5-3 min, 95% B; 3-3.2 min, 95-5% B | 3.5 | 0.3 | 5 | 2.15 | 2.14 |
| Monomethyl fumarate | Monomethyl fumarate-D3 | Waters ACQUITY UPLC BEH C18 (2.1 × 50 mm, 1.7 μm) | 25 | 0.01% acetic acid in water | 0.01% acetic acid in acetonitrile | 0-1 min, 2% B; 1-1.8 min, 2-95% B; 1.8-2.5 min, 95% B; 2.5-2.7 min, 95-2% B | 3 | 0.3 | 7.5 | 1.92 | 1.91 |
| Diazepam | Diazepam-D5 | Waters ACQUITY UPLC BEH C18 (2.1 × 50 mm, 1.7 μm) | 25 | 0.1% formic acid in water | 0.1% formic acid in acetonitrile | 0-2 min, 10-95% B; 2-3 min, 95% B; 3-3.2 min, 95-10% B | 3.5 | 0.3 | 5 | 1.96 | 1.95 |

**Table S8.** Simulated steady-state unbound and total drug concentrations/amounts in DRG, SN, SM, Br and SC with an assumed total plasma concentration of 100 ng/mL and the measured mean, K_p_, K_p,uu_ and K_p,uu,cell_, f_u,plasma_ and V_u,tissue_

| **Compound** | **Dorsal root ganglia (DRG)** | | | | | | | | | | | |
| --- | --- | --- | --- | --- | --- | --- | --- | --- | --- | --- | --- | --- |
|  | **Measured parameter** | | | | | **Simulated steady-state unbound and total drug concentration/amount** | | | | | | |
|  | K_p,DRG_ | K_p,uu,DRG_ | K_p,uu,cell,DRG_ | f_u,pl_ | V_u,DRG_  (mL/g DRG) | C_u,pl_ (ng/mL) | C_u,ISF,DRG_ (ng/mL) | C_u,ICF,DRG_ (ng/mL) | A_tot,DRG_ (ng/g DRG) | A_u,ISF,DRG_ (ng/g DRG) | A_u,cell,DRG_ (ng/g DRG) | A_b,cell,DRG_ (ng/g DRG) |
| Paclitaxel | 59 | 4.3 | 1.4 | 0.069 | 200 | 6.9 | 29 | 42 | 5865 | 5.9 | 34 | 5825 |
| Vincristine | 12 | 0.47 | 2.0 | 0.53 | 48 | 53.0 | 25 | 49 | 1191 | 5.0 | 39 | 1147 |
| Methotrexate | 0.41 | 1.1 | 0.94 | 0.39 | 0.94 | 39.0 | 44 | 41 | 41 | 8.7 | 33 | 0 |
| Nilotinib | 0.73 | 0.48 | 0.090 | 0.0065 | 237 | 0.7 | 0.31 | 0.028 | 73 | 0.06 | 0.022 | 73 |
| Isoniazid | 0.57 | 0.89 | 0.48 | 0.93 | 0.69 | 93.0 | 83 | 40 | 57 | 17 | 32 | 8.5 |
| Acrylamide | 0.90 | 1.2 | 0.43 | 0.96 | 0.76 | 96.0 | 118 | 51 | 90 | 24 | 41 | 26 |
| Varenicline | 6.0 | 2.2 | 1.8 | 0.92 | 3.0 | 92.0 | 199 | 352 | 600 | 40 | 281 | 279 |
| Oxycodone | 7.2 | 2.4 | 1.5 | 0.89 | 3.4 | 89.0 | 212 | 324 | 720 | 42 | 259 | 418 |
| Paroxetine | 24 | 0.51 | 0.94 | 0.086 | 555 | 8.6 | 4.4 | 4.1 | 2400 | 0.88 | 3.3 | 2396 |
| Monomethyl fumarate | 0.039 | 0.086 | 0.47 | 0.80 | 0.57 | 80.0 | 6.9 | 3.2 | 3.9 | 1.4 | 2.6 | 0 |
| Diazepam | 6.5 | 1.8 | 0.84 | 0.16 | 22 | 16.0 | 29 | 25 | 650 | 5.9 | 20 | 624 |
|  |  |  |  |  |  |  |  |  |  |  |  |  |
| **Compound** | **Sciatic nerve (SN)** | | | | | | | | | | | |
|  | **Measured parameter** | | | | | **Simulated steady-state unbound and total drug concentration/amount** | | | | | | |
|  | K_p,SN_ | K_p,uu,SN_ | K_p,uu,cell,SN_ | f_u,pl_ | V_u,SN_  (mL/g SN) | C_u,pl_ (ng/mL) | C_u,ISF,SN_ (ng/mL) | C_u,ICF,SN_ (ng/mL) | A_tot,SN_  (ng/g SN) | A_u,ISF,SN_ (ng/g SN) | A_u,cell,SN_ (ng/g SN) | A_b,cell,SN_ (ng/g SN) |
| Paclitaxel | 20 | 4.5 | 0.55 | 0.069 | 64 | 6.9 | 31 | 17 | 1980 | 6.2 | 14 | 1960 |
| Vincristine | 3.8 | 0.42 | 0.41 | 0.53 | 17 | 53.0 | 22 | 9.1 | 378 | 4.5 | 7.3 | 366 |
| Methotrexate | 0.46 | 1.2 | 0.90 | 0.39 | 1.0 | 39.0 | 46 | 42 | 46 | 9.2 | 33 | 3.6 |
| Nilotinib | 0.79 | 0.28 | 0.20 | 0.0065 | 427 | 0.7 | 0.18 | 0.036 | 79 | 0.036 | 0.029 | 79 |
| Isoniazid | 0.64 | 1.3 | 0.23 | 0.93 | 0.52 | 93.0 | 123 | 28 | 64 | 25 | 22 | 17 |
| Acrylamide | 0.61 | 0.58 | 0.11 | 0.96 | 1.1 | 96.0 | 56 | 6.1 | 61 | 11 | 4.9 | 45 |
| Varenicline | 3.3 | 1.2 | 1.9 | 0.92 | 3.0 | 92.0 | 109 | 207 | 330 | 22 | 166 | 143 |
| Oxycodone | 2.6 | 0.83 | 1.6 | 0.89 | 3.5 | 89.0 | 74 | 117 | 260 | 15 | 93 | 152 |
| Paroxetine | 11 | 0.19 | 0.91 | 0.086 | 649 | 8.6 | 1.6 | 1.5 | 1100 | 0.33 | 1.2 | 1098 |
| Monomethyl fumarate | 0.14 | 0.72 | 0.21 | 0.80 | 0.25 | 80.0 | 58 | 12 | 14 | 12 | 9.7 | 0 |
| Diazepam | 11 | 2.1 | 0.35 | 0.16 | 32 | 16.0 | 33 | 12 | 1100 | 6.7 | 9.4 | 1084 |
|  |  |  |  |  |  |  |  |  |  |  |  |  |
|  |  |  |  |  |  |  |  |  |  |  |  |  |
| **Compound** | **Skeletal muscle (SM)** | | | | | | | | | | | |
|  | **Measured parameter** | | | | | **Simulated steady-state unbound and total drug concentration/amount** | | | | | | |
|  | K_p,SM_ | K_p,uu,SM_ | K_p,uu,cell,SM_ | f_u,pl_ | V_u,SM_  (mL/g SM) | C_u,pl_ (ng/mL) | C_u,ISF,SM_ (ng/mL) | C_u,ICF,SM_ (ng/mL) | A_tot,SM_  (ng/g SM) | A_u,ISF,SM_ (ng/g SM) | A_u,cell,SM_ (ng/g SM) | A_b,cell,SM_ (ng/g SM) |
| Paclitaxel | 31 | 5.5 | 1.0 | 0.069 | 77 | 6.9 | 38 | 38 | 3096 | 7.6 | 30 | 3058 |
| Vincristine | 14 | 3.4 | 2.2 | 0.53 | 7.3 | 53.0 | 181 | 397 | 1389 | 36 | 317 | 1035 |
| Methotrexate | 0.13 | 0.27 | 1.0 | 0.39 | 1.2 | 39.0 | 11 | 11 | 13 | 2.1 | 8.4 | 2.5 |
| Nilotinib | 0.49 | 0.070 | 1.0 | 0.0065 | 1071 | 0.7 | 0.046 | 0.048 | 49 | 0.0091 | 0.038 | 49 |
| Isoniazid | 0.58 | 0.40 | 1.0 | 0.93 | 1.4 | 93.0 | 37 | 37 | 58 | 7.4 | 30 | 21 |
| Acrylamide | 0.69 | 0.22 | 1.0 | 0.96 | 3.0 | 96.0 | 21 | 21 | 69 | 4.2 | 17 | 48 |
| Varenicline | 4.1 | 0.83 | 2.2 | 0.92 | 5.3 | 92.0 | 76 | 171 | 413 | 15 | 137 | 261 |
| Oxycodone | 2.6 | 0.49 | 2.2 | 0.89 | 5.6 | 89.0 | 44 | 97 | 255 | 8.7 | 77 | 169 |
| Paroxetine | 14 | 0.41 | 2.2 | 0.086 | 373 | 8.6 | 3.5 | 7.9 | 1350 | 0.71 | 6.3 | 1343 |
| Monomethyl fumarate | 0.55 | 0.11 | 0.66 | 0.80 | 0.66 | 80.0 | 8.4 | 5.5 | 55 | 1.7 | 4.4 | 0 |
| Diazepam | 1.4 | 0.5 | 1.0 | 0.16 | 17 | 16.0 | 8.0 | 8.0 | 137 | 1.6 | 6.4 | 129 |
|  |  |  |  |  |  |  |  |  |  |  |  |  |
| **Compound** | **Brain (Br)** | | | | | | | | | | | |
|  | **Measured parameter** | | | | | **Simulated steady-state unbound and total drug concentration/amount** | | | | | | |
|  | K_p,Br_ | K_p,uu,Br_ | K_p,uu,cell,Br_ | f_u,pl_ | V_u,Br_  (mL/g Br) | C_u,pl_ (ng/mL) | C_u,ISF,Br_ (ng/mL) | C_u,ICF,Br_ (ng/mL) | A_tot,Br_  (ng/g Br) | A_u,ISF,Br_ (ng/g Br) | A_u,cell,Br_ (ng/g Br) | A_b,cell,Br_ (ng/g Br) |
| Paclitaxel | 1.7 | 0.032 | 11 | 0.069 | 745 | 6.9 | 0.22 | 2.5 | 166 | 0.044 | 2.0 | 164 |
| Vincristine | 0.24 | 0.00074 | 49 | 0.53 | 620 | 53.0 | 0.039 | 1.9 | 24 | 0.0078 | 1.5 | 22 |
| Methotrexate | 0.024 | 0.083 | 0.48 | 0.39 | 0.75 | 39.0 | 3.2 | 1.6 | 2.4 | 0.65 | 1.2 | 0.51 |
| Nilotinib | 0.069 | 0.014 | 0.54 | 0.0065 | 788 | 0.7 | 0.0088 | 0.0047 | 6.9 | 0.0018 | 0.0038 | 6.9 |
| Isoniazid | 0.67 | 1.0 | 0.20 | 0.93 | 0.69 | 93.0 | 97 | 19 | 67 | 19 | 15 | 32 |
| Acrylamide | 0.69 | 0.40 | 1.3 | 0.96 | 1.8 | 96.0 | 38 | 48 | 69 | 7.7 | 39 | 23 |
| Varenicline | 6.2 | 2.6 | 1.2 | 0.92 | 2.6 | 92.0 | 237 | 285 | 620 | 47 | 228 | 345 |
| Oxycodone | 3.3 | 0.94 | 1.6 | 0.89 | 4.0 | 89.0 | 84 | 137 | 330 | 17 | 110 | 204 |
| Paroxetine | 12 | 0.13 | 2.8 | 0.086 | 1058 | 8.6 | 1.1 | 3.1 | 1200 | 0.22 | 2.5 | 1197 |
| Monomethyl fumarate | 0.0092 | 0.044 | 0.19 | 0.80 | 0.26 | 80.0 | 3.5 | 0.67 | 0.92 | 0.70 | 0.54 | 0 |
| Diazepam | 3.8 | 1.2 | 0.67 | 0.16 | 19 | 16.0 | 20 | 13 | 380 | 4.0 | 11 | 365 |
|  |  |  |  |  |  |  |  |  |  |  |  |  |
| **Compound** | **Spinal cord (SC)** | | | | | | | | | | | |
|  | **Measured parameter** | | | | | **Simulated steady-state unbound and total drug concentration/amount** | | | | | | |
|  | K_p,SC_ | K_p,uu,SC_ | K_p,uu,cell,SC_ | f_u,pl_ | V_u,SC_ *  (mL/g SC) | C_u,pl_ (ng/mL) | C_u,ISF,SC_ (ng/mL) | C_u,ICF,SC_ (ng/mL) | A_tot,SC_  (ng/g SC) | A_u,ISF,SC_ (ng/g SC) | A_u,cell,SC_ (ng/g SC) | A_b,cell,SC_ (ng/g SC) |
| Paclitaxel | 1.3 | 0.026 | 11 | 0.069 | 745 | 6.9 | 0.18 | 2.0 | 132 | 0.036 | 1.6 | 130 |
| Vincristine | 0.45 | 0.0014 | 40 | 0.53 | 620 | 53.0 | 0.074 | 2.9 | 45 | 0.015 | 2.4 | 43 |
| Methotrexate | 0.04 | 0.12 | 0.75 | 0.39 | 0.75 | 39.0 | 4.8 | 3.6 | 4.0 | 0.97 | 2.9 | 0.13 |
| Nilotinib | 0.091 | 0.018 | 0.46 | 0.0065 | 788 | 0.7 | 0.012 | 0.0053 | 9.1 | 0.0023 | 0.0043 | 9.1 |
| Isoniazid | 0.51 | 0.79 | 0.55 | 0.93 | 0.69 | 93.0 | 73 | 41 | 51 | 15 | 32 | 3.7 |
| Acrylamide | 0.69 | 0.40 | 1.7 | 0.96 | 1.8 | 96.0 | 38 | 65 | 69 | 7.7 | 52 | 10 |
| Varenicline | 4.1 | 1.7 | 2.0 | 0.92 | 2.6 | 92.0 | 156 | 317 | 405 | 31 | 254 | 120 |
| Oxycodone | 2.9 | 0.80 | 2.0 | 0.89 | 4.0 | 89.0 | 71 | 145 | 286 | 14 | 116 | 156 |
| Paroxetine | 11 | 0.12 | 2.2 | 0.086 | 1058 | 8.6 | 1.1 | 2.4 | 1123 | 0.21 | 1.9 | 1121 |
| Monomethyl fumarate | 0.010 | 0.050 | 0.26 | 0.80 | 0.26 | 80.0 | 4.0 | 1.0 | 1.0 | 0.80 | 0.83 | 0 |
| Diazepam | 4.96 | 1.6 | 0.70 | 0.16 | 19 | 16.0 | 26 | 18 | 496 | 5.2 | 15 | 476 |

*V_u,SC_ is assumed to be the same as V_u,Br_

C_u,pl_: unbound plasma concentration

C_u,ISF,tissue_: Unbound drug concentration in tissue interstitial fluid

C_u,ICF,tissue_: Unbound drug concentration in tissue intracellular fluid

A_tot,tissue_: Total drug amount in tissue

A_u,ISF,tissue_: Unbound drug amount in tissue interstitial fluid, assuming the volume of extracellular space is 0.2 mL/g tissue

A_u,ICF,tissue_: Unbound drug amount in tissue intracellular fluid, assuming the volume of intracellular space is 0.8 mL/g tissue

A_b,cell,tissue_: Bound drug amount in the tissue parenchymal cells, calculated as A_tot,tissue_ – A_u,ISF,tissue_ – A_u,ICF,tissue_
