# Supplementary material for: Analysis of the contributing role of drug transport across biological barriers in the development and treatment of chemotherapy-induced peripheral neuropathy
Source: Fluids Barriers CNS. 2024 Feb 8;21:13. doi: 10.1186/s12987-024-00519-7 (PMC10854123; doi:10.1186/s12987-024-00519-7)
Supplement: Supplementary file 3 — Additional file 3. Supplementary Materials and Methods section | Extended Materials and Methods section. [file 12987_2024_519_MOESM3_ESM.docx]

**Supplementary Materials**

**Analysis of the Contributing Role of Drug Transport Across Biological Barriers in the Development and Treatment of Chemotherapy-Induced Peripheral Neuropathy**

Yang Hu^1,2^, Milda Girdenyté^1,3^, Lieke Roest^1^, Iida Liukkonen^1^, Maria Siskou^1^, Frida Bällgren^1^, Margareta Hammarlund-Udenaes^1^, and Irena Loryan^1,^*

^1^ Translational pharmacokinetics-pharmacodynamics group, tPKPD, Department of Pharmacy, Faculty of Pharmacy, Uppsala University, Box 580, SE-751 23, Uppsala, Sweden

^2^ Current affiliation: Discovery ADME, Drug Discovery Sciences, Boehringer Ingelheim RCV GmbH & Co KG, A-1121, Vienna, Austria

^3^ Pharmacy and Pharmacology Center, Institute of Biomedical Sciences, Faculty of Medicine, Vilnius University, M.K. Čiurlionio, Str. 21/27, LT-03101 Vilnius, Lithuania

* Corresponding author: irena.loryan@farmaci.uu.se

Irena Loryan, MD, PhD

Translational PKPD group, Department of Pharmacy

Box 580, 751 23 Uppsala, Sweden

+46 18 471 4995

[Irena.loryan@farmaci.uu.se](mailto:Irena.loryan@farmaci.uu.se)

# Extended materials and methods

Assessment of CIPN-site specific distribution was performed to characterize the extent of the transport across the blood-to-tissue barriers and parenchymal cellular barriers (CB) using key pharmacokinetic (PK) parameters, namely unbound tissue (extracellular)-to-plasma concentration ratio (K_p,uu_) and unbound intracellular-to-extracellular concentration ratio (K_p,uu,cell_), respectively (Fig. 1C). Herein, for the first time a novel Combinatory Mapping Approach-based methodology named CMA-CIPN was applied for the measurement of the parameters and mapping of the extent of distribution in conventional and non-conventional CIPN-sites including dorsal root ganglia (DRG), sciatic nerve (SN), brain (Br), spinal cord (SC), and skeletal muscle (SM). CMA-CIPN is consisting of *in vivo* pharmacokinetic studies and the set of *in vitro* assays for the evaluation of the uptake and the binding in the respective tissues. All the analytes in the samples were analyzed by ultra-performance liquid chromatography-tandem mass spectrometry (UPLC-MS/MS).

## Materials

Paclitaxel, paclitaxel-D5, vincristine sulfate, vincristine-D3 sulfate, nilotinib-D3, isoniazid-D4, varenicline tartrate, varenicline-D4 hydrochloride, monomethyl fumarate-D3 were ordered from Toronto Research Chemicals (Toronto, Canada). Methotrexate solution (1 mg/mL), methotrexate-D3 solution (100 μg/mL), nilotinib hydrochloride monohydrate, isoniazid, acrylamide, acrylamide-D3 solution (500 mg/L), paroxetine hydrochloride, paroxetine-D6 maleate solution (100 μg/mL), and monomethyl fumarate were purchased from Sigma-Aldrich (Stockholm, Sweden). Diazepam, oxycodone hydrochloride, paclitaxel Actavis injection solution (6 mg/mL), vincristine sulfate injection solution (1 mg/mL), and methotrexate injection solution (50 mg/mL) were obtained from Apoteket Production & Laboratories AB (Stockholm, Sweden). Oxycodone-D6 and Diazepam-D5 solution (1 mg/mL) were purchased from Cerilliant Corporation (Round Rock, TX, USA). Dimethyl sulfoxide (DMSO), ammonium acetate, and acetic acid were obtained from Sigma-Aldrich (Stockholm, Sweden). Acetonitrile (ACN), methanol, ammonia (25 %) and formic acid (98%) were obtained from Merck (Darmstadt, Germany). Ethanol absolute was obtained from VWR chemicals (VWR, Stockholm, Sweden). HEPES-buffered artificial extracellular fluid (aECF) were prepared in-house consisting of 129 mM NaCl, 10 mM glucose, 3 mM KCl, 1.4 mM CaCl_2_, 1.2 mM MgSO_4_, 0.4 mM K_2_HPO_4_, 25 mM HEPES, and 0.4 mM ascorbic acid. All chemicals and reagents used in experiments were of analytical grade. The water used was deionized in-house and purified with a Milli-Q Academic system (Millipore, Bedford, MA, USA).

## Animals

Male Sprague-Dawley rats (n = 91 in total) from Taconic (Lille Skensved, Denmark) or male Wistar-Han rats (n = 17 in total) from Charles River Laboratories, Inc. (Germany) were used for all the experiments. Before experiments, the rats were housed in groups and acclimatized for seven days under temperature- and humidity-controlled conditions in a 12 h light/dark cycle with unlimited access to food and water. The rats weighed 240-340 g on the day of the experiments. All the experimental protocols and animal procedures were approved by the Uppsala Regional Animal Ethics Committee (Dnr 5.8.18-12230/2019, Uppsala, Sweden) and were performed at the Department of Pharmacy, Biomedical Centre, Uppsala University (Husargatan 3, 751 23 Uppsala, Sweden). Animal studies have been reported in agreement with ARRIVE (Animal Research: Reporting of In Vivo Experiments) guidelines^1^. All studies were non-randomized and non-blinded. A priori estimated minimally required per group sample size for a two-tailed t-test study was six to four rats, given the probability level of α 0.05, the anticipated effect size, i.e., Cohen’s d in the range of 2 to 2.5, and the desired statistical power level of 0.8.

## *In vivo* pharmacokinetic studies to assess total drug distribution in CIPN-sites

The goal of this study was to estimate the total tissue-to-plasma concentration ratio, K_p,tissue_, by performing in vivo pharmacokinetic study in rats. Considering the potential time-dependent tissue partitioning, K_p,tissue_ of each drug were investigated under the steady-state condition following either a 4-h constant intravenous (IV) infusion or a continuous subcutaneous (SC) infusion up to 10 days via ALZET osmotic pump (NB: it was required only for paclitaxel and vincristine) in rats. The ALZET pumps were used due to the inability to achieve a steady-state in investigated tissues for paclitaxel and vincristine using a conventional 4-h IV infusion regimen, given the slow distribution and unparallel profiles between plasma and tissues (e.g., in DRG) reported earlier^2^. and in this study (Fig. S2).

For the IV infusion experiments, PE-50 catheters were surgically implanted one day before the experiments in the femoral artery and vein of the rats for blood sampling and drug administration, respectively. For isoniazid, monomethyl fumarate and oxycodone, a constant IV infusion from 0 to 4 h was given. For the other drugs, rats received a short (0.25 or 0.5 h) loading infusion followed by a constant infusion of up to 4 h. The initial loading infusion was implemented to achieve and maintain the targeted steady-state plasma concentration more rapidly. The dosing regimen of each drug was designed based on the rat PK parameters obtained from either the literature or a 10 min IV infusion PK study performed in-house and the targeted total plasma concentration that is clinically relevant, supported by the PK modeling and simulation using the Berkeley-Madonna software (version 8.3.18 for Windows, Berkeley, CA, USA). For the SC infusion experiments, ALZET osmotic pumps (model 2ML2, flow rate 5 μL/h) were loaded with drug solutions and implanted subcutaneously according to the manufacturer’s instructions. The dosing regimens (period, infusion rate), vehicles, drug concentration in the infusion solution, targeted steady-state total plasma concentration for each drug as well as number, sex, and species of rats per group are summarized in Supplementary Table S2.

Blood samples (~180 μL) were withdrawn from the arterial catheter at designated time points to examine the attainment of the steady-state. At the end of the experiments, the rats were anesthetized and terminal blood samples (minimum 10 mL) were collected via intracardiac puncture. Plasma was immediately obtained after centrifuging blood samples at 10000 rpm for 5 min. After decapitation, the brain, SN (pooled from right and left sides), SM (*m.* *biceps femoris*), SC (cervical and thoracic parts) and DRG (pooled from lumbar regions) were collected. All plasma and tissue samples after weighing were immediately frozen on dry ice and stored at -20 ^°^C. Before the sample analysis, the brain was homogenized in 1:4 (wet weight : volume) with phosphate buffer saline (PBS, pH 7.4) in two steps. First homogenization was performed mechanically using a Heidolph mechanical stirrer (Heidolph Instruments GmbH & Co, Schwabach, Germany) followed by ultrasonication for three cycles (5 s on and 5 s off) at an amplitude of 50 % using an ultrasonic processor VCX-130 (Sonics, Chemical Instruments AB, Stockholm, Sweden). SC (1:4, w:v), SM (1:9, w:v), SN (1:6 or 1:9, w:v), DRG (1:9, w:v) were homogenized with PBS using a 4-Place Beads Homogenizer (VWR, Stockholm, Sweden) at the maximal speed for 2, 4, 6 and 2 min, respectively, to assure complete homogenization of the small tissue pieces.

## Assessment of blood-to-tissue barrier integrity in CIPN-sites using a 4 kDa TRITC-dextran

The integrity of blood-to-tissue barriers was evaluated in healthy rats (n=3) using as a marker 4 kDa tetramethylrhodamine isothiocyanate (TRIC) dextran (TdB Consultancy AB, Uppsala, Sweden). One day before the experiment femoral vein and artery were catheterized for drug administration and blood sampling, respectively. The solution containing 50 mg/mL of 4 kDa TRITC-dextran in saline was administered at the dose of 400 mg/kg as a constant-rate intravenous infusion for 5 min using Harvard 22 pump (Harvard Apparatus Inc., Holliston, MA). Blood samples were taken at time 0 and 5 min from the start of the infusion. At the end of the dextran infusion, a blood sample was taken using the heart puncture technique followed by transcardial perfusion using 0.9 % NaCl for 2 min, at a rate of 10 mL/min. Before centrifugation, the blood was allowed to coagulate for 15-20 min at room temperature. The blood was then centrifuged at 10,000 rpm for 5 min. Thereafter, serum was collected, diluted ten-fold with MilliQ water and stored at 6 °C pending bioanalysis. CIPN-sites were weighted and collected in pre-weighted 2 mL (brain, SC, SM) and 0.5 mL (SN, DRG) tubes pre-filled with 2.8 mm and 1.4 mm ceramic beads (VWR® Soft Tissue Homogenizing Mix, VWR, Stockholm, Sweden), respectively. Tissues were homogenized in three (brain, SC, SM) and six (SN, DRG) volumes of MilliQ water using 4-Place Beads Homogenizer (VWR, Stockholm, Sweden). Samples were further centrifuged at 14.800 rpm for 1 h at 4 °C, and the supernatant was collected.

Standard curves in diluted serum (1:9, v:v, with MilliQ water) and the supernatant obtained from 1:3 blank brain homogenate (w:v with MilliQ water) were ranging from 5 - 1250 µg/mL and 0.05 – 365 µg/mL, respectively. Standards were prepared in blank serum and brain homogenate to reduce any impact of tissue autofluorescence in the samples. Quantification of dextran in the samples was performed within 4 hours after the end of the experiment. All necessary measures were taken to prevent any potential degradation of dextran due to light sensitivity.

Fifty µL of samples, standards and respective blanks were directly loaded onto 96-well microplates (solid black polystyrene, half area, flat bottom, Corning® Incorporated, Corning, NY, USA) in duplicates (except DRG where only one sample was obtained). The fluorescent intensity of TRITC-dextran was measured using a Tecan Spark® Multimode Microplate Reader (Tecan Group Ltd., Männedorf, Switzerland) at the excitation wavelength of 550 nm and emission wavelength of 571 nm. The signal was optimized by the well containing the highest standard.

## *In vitro* brain slice assay to evaluate drug brain tissue uptake and binding

The unbound volume of distribution of drug in brain (V_u,brain_) was assessed using the brain slice assay based on previously published protocols^3,4^. Briefly, three drug-naïve male Sprague-Dawley rats were used for each brain slice study per drug. The rats were sacrificed under isoflurane anesthesia, and the brain was collected and immersed in the ice-cold oxygenated artificial extracellular fluid (aECF).

Briefly, six 300 μm coronal slices from the striatum area were obtained from each rat brain using a vibrating blade microtome Leica VT1200 (Leica Microsystems AB, Sweden), and then transferred into a Ø80-mm flat-bottomed glass beaker with 15 mL of aECF containing tested drugs in a cassette, i.e., a mixture of up to five drugs in one beaker. A concentration range of 100-200 nM for each compound in a cassette is usually recommended, therefore, initial drug concentration in aECF of 100 nM for methotrexate, nilotinib, acrylamide, oxycodone and paroxetine and 200 nM for isoniazid, varenicline and diazepam was used ([4](#biblioRef03)). For paclitaxel and vincristine, buffer concentrations of 50 nM were chosen to avoid reaching the non-linear range associated with the saturation of the intracellular drug binding to tubulin which is above 100 nM for paclitaxel ([6](#biblioRef05)). A higher aECF concentration of 500 nM was needed for monomethyl fumarate to ensure that drug concentration in the slices was detectable due to the limited sensitivity of the analytical method and extremely low V_u,brain_ value.

A 5-h incubation was performed at 37 ^°^C in an orbital shaker MaxQ4450 (Thermo Fisher Scientific, NinoLab, Sweden) with a rotation speed at 45 rpm and constant oxygen supply at 75-80 mL/min, after which the buffer and brain slices were sampled. The buffer’s pH was immediately measured by the end of the incubation and was within the acceptable range, i.e., 7.4 ± 0.16 ([4](#biblioRef03)). When sampling, the buffer samples were mixed with an equal volume of blank brain homogenate (1:4, w:v with aECF) to match the matrix of the brain slice samples, i.e., 1:9 brain homogenate. At the end of the experiment, the brain slices were removed, dried on filter paper, weighed, and individually homogenized in aECF (1:9, w:v) with a VCX-130 ultrasonic processor (Sonics, Chemical Instruments AB, Sweden). The stability of each compound was evaluated in parallel with the brain slice experiments by sampling buffer samples from the scintillation vial without brain slices before and after the incubation. All samples were stored at −20 ^°^C before UPLC-MS/MS analysis.

By assuming that the drug concentration in the protein-free buffer is equal to that in the interstitial fluid of the brain slice at equilibrium, the V_u,brain_ (mL/g brain) was calculated using Eq. 1 as a ratio of the drug amount in the brain slice (A_slice_, nmol/1000 g brain) to the measured buffer concentration (C_buffer_, nmol/L) at the end of the incubation. The density of brain tissue was assumed to be 1 g/mL.

|  | $V_{u,brain}= \frac{A_{slice} -C_{buffer} \times V_{i}}{C_{buffer} \times(1- V_{i})}$ | (1) |
| --- | --- | --- |

Where V_i_ (mL/g brain) represents the volume of buffer layer surrounding brain slices due to incomplete buffer absorption by the filter paper. A V_i_ of 0.133 mL/g brain obtained using the marker ^14^C-sucrose was used in the calculations. V_i_ was assessed in three independent experiments where six 300 µm rat brain slices were incubated in 15 mL aECF containing 500 nM ^14^C-sucrose (4.35 μCi/0.16 MBq) for 15, 45 or 90 s in three glass beakers (each time point per beaker). Brain slices were collected and weighed in scintillation vials at each time point (3 samples per time point, i.e., due to low concentration of ^14^C-sucrose in the brain slices two slices were pooled). One buffer sample was taken from each beaker at each time point after the slices were collected. After collecting the tissue samples, 1 mL of tissue solubilizer Solvable was added to each sample and the mixture was incubated overnight at 50 °C on the orbital shaker at 100 rpm. After complete tissue solubilization, 15 mL of Ultima Gold liquid scintillation cocktail was added to each sample on the day of the radioactivity measurements. All measurements were performed on a TRI-CARB 4910TR liquid scintillation counter (PerkinElmer, Waltham, MA, USA). Total radioactivity was measured in tissues (A_tissue_, Becquerel/g tissue) and buffer (C_buffer_, Becquerel/mL buffer). The ratio A_tissue_/C_buffer_ (milliliters per gram of slice) was plotted against time (seconds), and linear regression analysis was used to calculate a zero-time y-intercept. Since there is no uptake of ^14^C-sucrose into the tissue at time zero, the extrapolated A_tissue_/C_buffer_ ratio at time zero represents V_i_, i.e., the volume of the buffer film that covers the sampled tissue.

The spiking recovery was calculated according to Eq. 2 to examine if the initial concentration of each compound deviated from the theoretical level due to poor solubility in aECF or sticking to the scintillation vial.

|  | $Spiking recovery \left( \% \right)= \frac{C_{0h}}{C_{theoretical}}\times100\%$ | (2) |
| --- | --- | --- |

Where C_0h_ and C_theoretical_ are the measured and theoretical concentrations in the aECF before the incubation (at 0 h), respectively.

The thermostability of the drugs during the incubation was calculated based on Eq. 3. A thermostability within 100 ± 30 % was considered acceptable ([7](#biblioRef06))

|  | $Thermostability as \%remaining= \frac{C_{5h}}{C_{0h}}\times100\%$ | (3) |
| --- | --- | --- |

Where C_0h_ and C_5h_ are the measured buffer concentrations from the scintillation vials before and after 5-h incubation, respectively.

## *In vitro* peripheral nervous tissue assay to estimate drug DRG and SN uptake and binding

The unbound volume of distribution of drug in DRG (V_u,DRG_) or SN (V_u,SN_) was assessed using a novel approach by adapting brain slice assay to the smaller size neural tissues such as DRG and SN (Fig. 5A). Scaling down of brain slice assay to smaller PNS tissues was validated by using three punched small pieces of brain tissue from 500 µm brain slice (two pieces were taken from cortical and one from striatal area) weighing ca 10 mg to match expected weight of DRG and SN. Paclitaxel and paroxetine were selected as model drugs for the optimization of the experimental conditions as their intra-tissue distribution is governed by mechanisms impacting the time to reach the equilibrium, namely specific binding to tubulin for paclitaxel and lysosomal trapping for paroxetine. Obtained V_u,brain_ values for model drugs were compared to those obtained from the standard brain slice assay (See Section 5). Experimental conditions assuring achievement of similar V_u,brain_ values obtained by conventional brain slice assay were applied in further analysis.

This newly developed method featured the incubation of drugs either in the cassette form in a 24-well plate with DRG or SN freshly collected from drug-naïve rats (standard) or incubation of blank aECF with DRG or SN collected from rats receiving the drug *in vivo*. The latter was performed only for paclitaxel, paroxetine and nilotinib to facilitate the attainment of steady-state within the 5 h incubation.

For cassette 1, three drug-naïve male Sprague-Dawley rats were sacrificed under isoflurane anesthesia. DRG and SN (both sides) were collected and immersed in 500 μL ice-cold oxygenated aECF containing aa 100 nM isoniazid, varenicline, oxycodone and methotrexate, and aa 1000 nM acrylamide and monomethyl fumarate. For cassette 2, at least three drug-naïve male Sprague-Dawley rats initially received 10 min IV infusion of paclitaxel, paroxetine and nilotinib at 10, 1 and 2.5 mg/kg, respectively. Then, DRG and SN (both sides) were collected and immersed in 500 μL oxygenated aECF containing 50 nM vincristine and 200 nM diazepam. Each well contained on average 10 mg of DRG (five lumbar DRG) or SN (left or right), enabling two technical replicates from one biological replicate to be included. A 5 h incubation was performed at 37 ^°^C in an orbital shaker MaxQ4450 (Thermo Fisher Scientific, NinoLab, Sweden) with a rotation speed at 100 rpm and constant oxygen supply at 75-80 mL/min, after which the buffer and tissues were sampled.

At the end of incubation, 50 μL sample was taken from the buffer. DRGs and SN were individually removed, weighed and homogenized in aECF (1:9, w:v) using a 4-Place Beads Homogenizer (VWR, Stockholm, Sweden) at the maximal speed for 2 and 6 min, respectively. Each compound’s spiking recovery and thermostability were evaluated in parallel by incubating 500 μL drug-containing aECF without tissues in a well (in duplicates) followed by a collection of buffer samples (50 μL) from the wells before and after the incubation. All samples were stored at −20 ^°^C before UPLC-MS/MS analysis.

With the assumption that at equilibrium the drug concentration in the protein-free buffer is equal to that in the interstitial fluid of DRG and SN, the V_u,DRG_ (mL/g DRG) and V_u,SN_ (mL/g SN) were calculated using Eq. 4 as a ratio of the drug amount in the DRG (A_DRG_, nmol/1000 g DRG) or SN (A_SN_, nmol/1000 g SN) to the measured buffer concentration (C_buffer_, nmol/L) at the end of the incubation. The density of DRG and SN was assumed to be 1 g/mL.

|  | $V_{u,DRG or SN}= \frac{A_{DRG or SN} -C_{buffer} \times V_{i}}{C_{buffer} \times(1- V_{i})}$ | (4) |
| --- | --- | --- |

Where V_i_ (mL/g DRG or SN) is the volume of the layer of aECF surrounding DRG or SN. Vi mean values of 0.146 and 0.122, measured individually in an independent study using the marker ^14^C-sucrose, were used to calculate V_u,SN_ and V_u,DRG_, respectively. V_i_ was assessed in three independent experiments in a similar to the determination of V_i_ in the brain slices manner (See Section 5). Briefly, SN (two strips of SN ~10 mg) and DRG (five DRG ~10 mg) from drug naïve rats were incubated in 500 μL aECF containing 500 nM ^14^C-sucrose for 15, 30, 60 or 90 s in each well in a 24-well plate. At designated time points SN and DRG from each well were collected and weighed in scintillation vials. One buffer sample (50 μL) containing ^14^C-sucrose was taken from each well at each time point after collection of SN and DRG. Total radioactivity and V_i_ assessment was performed as it is described in Section 5. The spiking recovery and thermostability were evaluated using a similar to the brain slice approach (See Section 5, Eq. 2, 3).

## *In vitro* equilibrium dialysis to assess drug plasma protein and tissue binding

The fraction of unbound drug in rat undiluted plasma, neural and skeletal muscle tissue homogenates was assessed by equilibrium dialysis (ED). Blank brain, SC, SM, SN and DRG collected from at least three drug-naïve male Sprague-Dawley rats were homogenized 1:9 (w:v) with PBS, pH 7.4 using a 4-Place Mini Beads Homogenizer (VWR, Stockholm, Sweden). The plasma and tissue homogenates were stored in aliquots at −80 ^°^C pending use.

One day before the ED experiment, dialysis membrane strips with a molecular weight cutoff of 12–14 kDa (HTDialysis LLC, Gales Ferry, CT, USA) were conditioned in PBS for 1 h and subsequently soaked overnight in a mixture of ethanol:PBS (20:80, v:v). On the day of the experiment, the dialysis membranes were rinsed with PBS. A Teflon 96-well ED device (HTDialysis LLC, Gales Ferry, CT, USA) was assembled based on the manufacturer’s instructions ([8](#biblioRef07)). The frozen tissue homogenates and undiluted plasma were thawed at room temperature. The pH of plasma was adjusted to 7.3 with 1 mol/L phosphoric acid. The thawed tissue homogenate and acidified plasma were spiked with the drugs of interest in Eppendorf tubes to a final concentration of 1 µM or 5 µM (only for nilotinib). An aliquot of 100 μL undiluted plasma or tissue homogenate from three biological replicates spiked with drugs of interest was added in duplicates (for each biological replicate) to one side of the dialysis membrane (donor side) and an equal volume of PBS was added to the other side of the membrane (receiver side). The ED device was incubated for 6 h (4 h for acrylamide and isoniazid due to the stability issue identified in a pilot study, data not shown) at 37 °C and 200 rpm using a MaxQ4450 orbital shaker (Thermo Scientific, Waltham, MA, USA). The remaining spiked plasma or tissue homogenate in Eppendorf tubes was also incubated alongside the ED device. In order to prevent pH changes and evaporation, the adhesive sealing film was used to cover the samples.

At the end of incubation, aliquots of 50 µL spiked tissue homogenate or plasma samples were taken from the donor chambers and added into a Corning^®^ 0.33 mL round-bottom polypropylene 96-well plate (VWR, Stockholm, Sweden) containing 50 µL of PBS. Aliquots of 50 µL dialyzed PBS samples from the receiver chambers were collected and mixed with 50 µL of blank plasma or respective tissue homogenate (1:9, w:v with PBS) in the same 96-well plate. Due to the limited volume of blank DRG homogenate, blank brain homogenate was used as a surrogate, since the use of deuterated internal standards could compensate for any potential matrix effect occurring during UPLC-MS/MS analysis. Before and after the incubation, aliquots of 50 µL of spiked plasma or tissue homogenate incubated were sampled from the Eppendorf tubes placed alongside the ED device and mixed with 50 µL of PBS in the sampling 96-well plate. These samples were collected to evaluate the recovery and stability. The 96-well plate was sealed with aluminum film, vortexed and stored at −20 °C pending bioanalysis.

The unbound fraction of each drug in plasma (f_u,plasma_) was calculated as:

|  | $f_{u,plasma}= \frac{C_{buffer}}{C_{plasma}}$ | (5) |
| --- | --- | --- |

Where C_buffer_ and C_plasma_ are the drug concentration determined in the buffer (receiver side) and plasma (donor side) samples, respectively.

The unbound fraction of each drug in the diluted (D) tissue homogenate (f_u,hD_) was calculated as:

|  | $f_{u,hD}= \frac{C_{buffer}}{C_{homogenate}}$ | (6) |
| --- | --- | --- |

Where C_buffer_ and C_homogenate_ represent the concentration determined in the buffer (receiver side) and tissue homogenate (donor side) samples, respectively.

Due to dilution, f_u,hD_ is often higher than the actual f_u,tissue_. As a result, f_u,D_ was corrected for the dilution factor (D, 10 in this case) to obtain the actual f_u,tissue_, as described in Eq. 7 ([9](#biblioRef08)).

|  | $f_{u,tissue}= \frac{f_{u,hD}}{{D+f_{u,hD}-D\times f}_{u,hD}}$ | (7) |
| --- | --- | --- |

The spiking recovery was calculated according to Eq. 8 to examine if the initial concentration of each compound spiked in plasma or tissue homogenate was similar to the theoretical level.

|  | $Spiking recovery \left( \% \right)= \frac{C_{0h}}{C_{theoretical}}\times100\%$ | (8) |
| --- | --- | --- |

Where C_0h_ and C_theoretical_ are the measured and theoretical concentrations in the spiked plasma or tissue homogenate before the incubation (at 0 h), respectively.

The ED recovery (mass balance) of each compound was calculated based on Eq. 9 . A recovery within 100 ± 30 % was arbitrarily considered acceptable^5^.

|  | $ED recovery \left( \% \right)= \frac{C_{donor}\times V_{donor}+C_{receiver}\times V_{receiver}}{C_{0h}\times V_{donor}}\times100\%$ | (9) |
| --- | --- | --- |

Where C_donor_ is the measured concentration in the donor side samples. C_receiver_ is the measured concentration in the receiver side samples. C_0h_ represents the measured concentration in the spiked plasma or tissue homogenate before the incubation.

The stability of the drugs during the incubation was calculated based on Eq. 10. A recovery within 100 ± 30 % was considered acceptable^5^.

|  | $Stability as \%remaining= \frac{C_{5h}}{C_{0h}}\times100\%$ | (10) |
| --- | --- | --- |

Where C_0h_ and C_5h_ are the measured concentrations in the spiked plasma or tissue homogenate before and after the 5 h incubation, respectively.

## Bioanalysis

Quantitative analysis of all the drugs in plasma and tissue homogenate samples was carried out using UPLC-MS/MS. Chromatographic separation of compounds was achieved using a Waters ACQUITY UPLC system that consists of a binary solvent manager (pumps), a sample manager (autosampler), a sample organizer and a column heater (Waters Corporation, Milford, MA, USA). The column used was either an ACQUITY UPLC BEH C18 column (2.1 × 50 mm, 1.7 μm) or an ACQUITY UPLC HSS C18 column (2.1 × 100 mm, 1.8 μm) protected by an ACQUITY UPLC BEH C18 VanGuard pre-column (2.1 × 5 mm, 1.7 μm) (Waters Corporation, Milford, MA, USA). The MS/MS detection was performed by multiple reaction monitoring using a Waters Xevo TQ-S Micro triple quadrupole mass spectrometer equipped with an electrospray ionization (ESI) source (Waters Corporation, Milford, MA, USA). System control and data processing were conducted using MassLynx 4.2 software (Waters Corporation, Milford, MA, USA). The details of sample preparation procedures and LC-MS/MS conditions for each analyte are shown in Supplementary Tables S4, S5 and S6. A suitable dynamic range was attained for all analytes. Standards (minimum six levels) and quality-control (three levels) samples were prepared in respective matrices. The lowest non-zero standard in the calibration curve was defined as the lower limit of quantification (LLOQ). Blanks, calibration curves and quality controls were included in all analytical runs. All standard curves were accepted at R^2^ values of ≥0.99.

## PK parameters mapping the extent of transport of unbound drug across endothelial and parenchymal cellular barriers

The total tissue-to-plasma concentration ratio at steady-state (K_p,tissue_) for each rat was calculated for the five investigated tissues using Eq. 11.

|  | $K_{p,tissue}= \frac{C_{tot,tissue,ss}}{C_{tot,plasma,ss}}$ | (11) |
| --- | --- | --- |

Where C_tot,tissue,ss_ represents the measured total drug concentration in DRG, SN, Br, SC or SM at the end of IV or SC infusion. C_tot,plasma,ss_ is the determined total steady-state drug concentration in plasma at the same time point.

To assess the extent of drug transport at DRG, SN, Br, SC or SM endothelial barriers, the unbound tissue extracellular-to-plasma concentration ratio (K_p,uu_) was calculated according to Eq. 12 ^6^.

|  | $K_{p,uu}= \frac{K_{p,tissue}}{V_{u,tissue}\times f_{u,plasma}}$ | (12) |
| --- | --- | --- |

Where V_u,tissue_ is the unbound volume of distribution of a drug in DRG, SN or brain estimated using the *in vitro* peripheral tissue distribution or brain slice assay. V_u,SC_ is assumed to be the same as V_u,brain_, given the similarity between brain and spinal cord drug tissue binding properties with a correlation coefficient of 0.96 (Supplementary Figure S4). V_u,SM_ was assessed using an inverse relationship between f_u_ and V_u_, corrected for the pH partitioning as described by Friden et al. ^7^. f_u,plasma_ is the unbound fraction of drug in plasma measured by ED.

To evaluate the extent of drug transport at DRG, SN and Br parenchymal cellular barriers, the unbound intracellular-to-extracellular concentration ratio (K_p,uu,cell_) was calculated based on Eq. 13 ^8^.

|  | $K_{p,uu,cell}= V_{u,tissue}\times f_{u,tissue}$ | (13) |
| --- | --- | --- |

f_u,tissue_ is the unbound fraction of drug in DRG, SN or brain measured by ED.

## Statistical analysis

Statistical analyses were performed using GraphPad Prism 9.5.0 for Windows (GraphPad Software, San Diego, CA, USA). The differences between K_p,uu,tissue_, K_p,uu,cell,tissue_, V_u,tissue_ and f_u,tissue_ were evaluated by a one-way ANOVA test followed by Tukey’s multiple comparisons if the dataset was tested to be normally distributed. If normal distribution did not apply to the dataset, Kailas-Kruskal test followed by Dunn’s multiple comparisons was performed. A *p* < 0.05 suggests a significant difference. Data are expressed as the mean ± standard deviation (SD).

The SD for K_p,uu_ and K_p,uu,cell_ was calculated following the law of propagation of error since they were derived from three (Eq. 12) or two (Eq. 13) parameters with uncertainty around the mean of each parameter ^9^. Propagation of uncertainty was estimated for both product and quotient of two variables, A and B, using the following equations.

Propagation of uncertainty of K_p,uu,cell_ was calculated according to the product rule. Let A and B be variables with respective SD σ_A_ and σ_B_ and set

|  | $f= A\cdot B$ | (16) |
| --- | --- | --- |

Propagation uncertainty for a product, i.e., the SD of *f*, was then calculated as follows:

|  | $\sigma_{f}\approx\left\vert f \right\vert\times\sqrt{\left( \frac{\sigma_{A}}{A} \right)^{2}+\left( \frac{\sigma_{B}}{B} \right)^{2}+2\frac{\sigma_{AB}}{AB}}$ | (17) |
| --- | --- | --- |

The covariance σ_AB_ was calculated with the correlation r as σ_AB_ = rσ_A_σ_B_

Propagation of uncertainty of K_p,uu_ was calculated according to the quotient rule. Let A and B be variables with respective SD σ_A_ and σ_B_ and set

|  | $f= \frac{A}{B}$ | (18) |
| --- | --- | --- |

Propagation uncertainty for a quotient, i.e., the SD of f, was then calculated as follows:

|  | $\sigma_{f}\approx\left\vert f \right\vert\times\sqrt{\left( \frac{\sigma_{A}}{A} \right)^{2}+\left( \frac{\sigma_{B}}{B} \right)^{2}-2\frac{\sigma_{AB}}{AB}}$ | (19) |
| --- | --- | --- |

As abovementioned, the covariance was calculated as σ_AB_ = rσ_A_σ_B_. Considering the innate correlation between the variables, |r| = 0.5 was assumed in all formulas. A negative correlation, i.e., r = −0.5, is present between V_u,tissue_ and f_u,plasma_, and f_u,tissue_ while all other parameters were positively correlated, i.e., r = 0.5.

## **References**

1. Percie du Sert, N.*, et al.* Reporting animal research: Explanation and elaboration for the ARRIVE guidelines 2.0. *PLoS biology* **18**, e3000411 (2020).

2. Nakamura, I.*, et al.* An in vivo mechanism for the reduced peripheral neurotoxicity of NK105: a paclitaxel-incorporating polymeric micellar nanoparticle formulation. *Int J Nanomedicine* **12**, 1293-1304 (2017).

3. Fridén, M.*, et al.* Development of a high-throughput brain slice method for studying drug distribution in the central nervous system. *Drug metabolism and disposition: the biological fate of chemicals* **37**, 1226-1233 (2009).

4. Loryan, I., Friden, M. & Hammarlund-Udenaes, M. The brain slice method for studying drug distribution in the CNS. *Fluids Barriers CNS* **10**, 6 (2013).

5. Di, L., Umland, J.P., Trapa, P.E. & Maurer, T.S. Impact of recovery on fraction unbound using equilibrium dialysis. *J Pharm Sci* **101**, 1327-1335 (2012).

6. Loryan, I.*, et al.* Mechanistic understanding of brain drug disposition to optimize the selection of potential neurotherapeutics in drug discovery. *Pharm Res* **31**, 2203-2219 (2014).

7. Fridén, M.*, et al.* Measurement of unbound drug exposure in brain: modeling of pH partitioning explains diverging results between the brain slice and brain homogenate methods. *Drug metabolism and disposition: the biological fate of chemicals* **39**, 353-362 (2011).

8. Fridén, M., Gupta, A., Antonsson, M., Bredberg, U. & Hammarlund-Udenaes, M. In vitro methods for estimating unbound drug concentrations in the brain interstitial and intracellular fluids. *Drug metabolism and disposition: the biological fate of chemicals* **35**, 1711-1719 (2007).

9. Taylor, J.R. An Introduction to Error Analysis: The Study of Uncertainties in Physical Measurements. (1997).
